# Molecular Surface Engineering of Sulfide Electrolytes with Enhanced Humidity Tolerance for Robust Lithium Metal All‐Solid‐State Batteries

**DOI:** 10.1002/adma.202515013

**Published:** 2025-12-16

**Authors:** Laras Fadillah, Leonie Braks, Jihoon Oh, Mingliang Liu, Hanna Türk, Davide Tisi, Mounir Mensi, Michele Ceriotti, Jang Wook Choi, Ali Coskun

**Affiliations:** ^1^ Department of Chemistry University of Fribourg Fribourg 1700 Switzerland; ^2^ School of Chemical and Biological Engineering and Institute of Chemical Process Seoul National University Seoul 08826 South Korea; ^3^ Ecole Polytechnique Fédérale de Lausanne Institute of Materials Lausanne 1015 Switzerland; ^4^ École Polytechnique Fédérale de Lausanne Institute of Chemical Sciences and Engineering (ISIC) Sion 1950 Switzerland

**Keywords:** argyrodite solid electrolyte, interfacial stability, organic coatings, solid‐state batteries

## Abstract

Solid‐state electrolytes (SSEs) enable next‐generation batteries due to their intrinsic safety and compatibility with lithium (Li) metal anodes. However, many SSEs, particularly sulfide‐based systems, suffer from limited electrochemical stability and high moisture sensitivity. Here, the molecular surface engineering of Li argyrodite SSE, Li_6_PS_5_Cl_0.5_Br_0.5_ (LPSClBr), is reported using octadecyl phosphonic acid (OPA) and its lithiated form (Li‐OPA) in a single‐step coating strategy to stabilize both anode and cathode interfaces. The Li‐OPA‐coated electrolyte maintains high ionic conductivity (>2.5 mS cm^−1^) and retains >92% of its initial conductivity after 24 h dry room exposure (dew point −50 °C). At 2 wt.% loading, Li‐OPA‐coated LPSClBr achieves a critical current density of 2.4 mA cm^−1^ and supports stable Li plating/stripping for over 400 h at 1.0 mAh cm^−2^. In NCM811 cathode‐based all‐solid‐state cells, it delivers 160 mAh g^−1^ at 0.3 C with >99.7% Coulombic efficiency and 85% capacity retention after 100 cycles. In anode‐free cell configurations, Li‐OPA‐modified electrolytes enhance interfacial stability and cycling performance. These results demonstrate Li‐OPA as a scalable, high‐performance interfacial modifier for sulfide‐based solid‐state batteries.

## Graphical abstract

It is demonstrated that the electrochemical, interfacial, and humidity stability of halide‐doped sulfide electrolytes (LPSClBr) is significantly enhanced by an organic surface coating using octadecyl phosphonic acid (OPA) and its lithiated form (Li‐OPA). This single‐step strategy enables robust interfacial protection, supports lithium metal anodes, and improves long‐term performance in high‐voltage all‐solid‐state batteries, including anode‐free configurations.

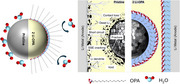

## 1 Introduction

The growing global demand for safer and higher‐performance energy storage systems has established all‐solid‐state batteries (ASSBs) as a promising solution for next‐generation energy technologies. ASSBs hold the promise of enabling lithium (Li) metal anodes, which offer significant advantages, including the high theoretical specific capacity (3860 mAh g^−1^) and low reduction potential (−3.04 V vs S.H.E),^[^ ^1^ ^]^ and can impart enhanced safety, higher energy density, and greater thermal stability compared to conventional liquid‐electrolyte batteries. Among solid‐state electrolytes (SSEs), argyrodite‐type sulfide‐based electrolytes stand out due to their exceptional ionic conductivity, ≈10^−3^ S cm^−1^, comparable to that of liquid organic electrolytes. However, their practical application faces major challenges, the mismatch between the electrochemical stability window of sulfide SSEs (1.71 ‐ 2.31 V vs Li⁺/Li)^[^ ^2^, ^3^ ^]^ with the operating voltage range of Li metal anode and high‐voltage cathodes such as LiCoO_2_ and Li(Ni*_x_* Mn*_y_* Co*_z_*)O_2_ (*x*+*y*+*z* = 1)^[^ ^4^, ^5^ ^]^ leads to interfacial degradation and consequent capacity loss. At high voltages, sulfide electrolytes electrochemically oxidize during charge, forming P_2_S*_x_* (*x* > 5), polysulfide (‐S‐S‐), and oxidized sulfur/phosphorus species at the interface between cathode and solid electrolyte.^[^ ^6^, ^7^, ^8^ ^]^ In addition, the P─S bond in LPSCl is thermodynamically less stable than the P─O bond, rendering sulfide‐based electrolytes highly reactive.^[^ ^9^, ^10^ ^]^ They not only react with oxide‐based cathode materials, but also with moisture, leading to the formation of phosphates and oxysulfides on the SSE surface. Their extreme sensitivity to moisture, even in a practical dry room environment, necessitates stringent handling and storage procedures since minor exposure to humidity triggers structural degradation and releases toxic by‐products Equation (1),^[^ ^11^ ^]^ as seen in the following reaction:

(1) $$Li_{6}PS_{5}Cl+4H_{2}O\;\to \;LiCl+Li_{2}S+Li_{3}PO_{4}+4H_{2}S↑$$

Addressing these interfacial and stability challenges has driven extensive research into interfacial engineering strategies. Protective interlayers like Li_3_N,^[^ ^12^, ^13^ ^]^ Li_7_N_2_I,^[^ ^14^ ^]^ LiF,^[^ ^15^, ^16^ ^]^ and Mg_16_Bi_84_,^[^ ^17^ ^]^ Li_3_Y_1−x_Zr_x_Cl_6_, Li_6_PS_5_Cl:B^[^ ^18^ ^]^ have been introduced to minimize interfacial resistance and suppress Li dendrite formation. Additionally, alloying Li metal with different metals, forming intermetallics such as Li‐In,^[^ ^19^ ^]^ Li‐Al,^[^ ^20^ ^]^ Li‐Ag,^[^ ^21^ ^]^ or Li‐Mg^[^ ^22^ ^]^ helps stabilize the anode interface during cycling. On the cathode side, protective coatings such as LiNbO_3_ (LNO),^[^ ^23^ ^]^ Li_4_Ti_5_O_12_,^[^ ^24^, ^25^ ^]^ Li_3‐x_B_1‐x_C_x_O_3_ (LBCO),^[^ ^26^ ^]^ Li_3_InCl_6_ (LIC),^[^ ^27^ ^]^ Li_2_O‐ZrO_2_ (LZO),^[^ ^28^ ^]^ and LiBF_4_ (LBF)^[^ ^29^ ^]^ have been applied to cathode active materials (CAMs) to suppress unwanted side reactions and enhance cycling stability. However, such coatings could crack or detach during volume change of the cathode and significantly alter the charge transport properties of the cathode materials.^[^ ^10^, ^30^, ^31^ ^]^ Structural modifications through halogen doping in the argyrodite electrolyte structure (Li_6_PS_5_ *X*) with elements like chlorine (Cl), bromine (Br), and iodine (I) have successfully improved ionic conductivity and widened the electrochemical stability window.^[^ ^32^, ^33^ ^]^ Mixed halogen doping, such as Li_6_PS_5_Cl_0.5_Br_0.5_ (LPSClBr), further enhances electrochemical properties by balancing structural stability and ionic transport.^[^ ^34^ ^]^

In parallel, polymeric and organic small‐molecule coatings have emerged as a promising strategy for stabilizing the interfaces.^[^ ^35^ ^]^ Polydimethylsiloxane (PDMS)^[^ ^36^ ^]^ and thiols such as 1‐undecanethiol (UDSH), 2‐(trimethylsilyl)ethanethiol can adsorb onto the electrolyte surface, forming a hydrophobic shield.^[^ ^37^, ^38^ ^]^ Lewis acid additives such as aluminum acetylacetonate (acaca), triphenyl carbenium tetrafluoroborate (tpctfb), tritylium hexafluorophosphate (tthfp), Li^+^‐conductive superhydrophobic protection layer with Li_1.4_Al_0.4_Ti_1.6_(PO_4_)_3_ (LATP) nanoparticles, and fluorinated polysiloxane (F‐POS), have been shown to suppress the reaction of LPSCl electrolyte by creating a water impermeable layer.^[^ ^39^, ^40^ ^]^ Succinonitrile, lithium difluoro(oxalato)borate (LiDFOB), and octadecyl phosphonic acid (OPA) have shown promise in enhancing the interfacial durability and cycling stability of CAMs.^[^ ^41^, ^42^ ^]^ Recently, our group has demonstrated that lithiated trithiocyanuric acid (Li_3_TCA)‐coated LPSCl exhibits remarkable compatibility with Li metal, enabling Li | NbO‐NCM811 full cells to operate for over 500 cycles at 0.3C.^[^ ^43^ ^]^ However, long‐term cycling stability of LPSCl‐based solid electrolytes still heavily depends on applying separate interfacial modifications at both the anode and the cathode, which adds complexity and manufacturing cost, thus highlighting the critical need for a more integrated and scalable stabilization strategy.

Here, we report the surface functionalization of LPSClBr using octadecyl phosphonic acid (OPA) and its lithiated derivative (Li‐OPA) to form a chemically and electrochemically stable interface that significantly suppresses the moisture sensitivity and mitigates the interfacial degradation of LPSClBr at both the cathode and anode interface. This electrolyte‐centered strategy enhances interfacial compatibility and simplifies ASSB design by eliminating the need for separate electrode coatings, offering a more practical and scalable approach. OPA serves as an effective organic surface modifier, where its long alkyl chain imparts hydrophobicity that protects the electrolyte from moisture ingress, while the polar phosphonic acid headgroup anchors on the surface of LPSClBr via noncovalent interactions with surface‐exposed sulfur and lithium species. This promotes spatially confined and stable surface functionalization. Furthermore, Li‐OPA facilitates Li‐ion transport, enhancing compatibility of the solid electrolyte with Li metal while preserving ionic conductivity across the interface. Electrochemical tests, including electrochemical impedance spectroscopy (EIS), cyclic voltammetry (CV), and full‐cell measurements using NMC811 cathodes demonstrate that 2 Li‐OPA (2 wt% of lithiated OPA and 98 wt% of LPSClBr) achieves a critical current density of 2.4 mA cm^−2^ at 1 mAh cm^−1^, rate capability, and long‐term cycling stability. In an NMC811|LPSClBr full‐cell configuration, 2 Li‐OPA delivers a discharge specific capacity of 160 mAh g^−1^ at 0.3C with over 99.7% Coulombic efficiency (CE) after 250 cycles, even without any pre‐NMC particle coating. Furthermore, Li‐OPA effectively mitigates structural degradation caused by moisture exposure in a practical dry room environment (dew point −50 °C) for 24 h. These results highlight Li‐OPA as a scalable, high‐performance interfacial material for enabling next‐generation ASSBs with Li metal and high‐voltage cathodes.

## 2 Results and Discussion

### 2.1 Interfacial Chemistry of OPA and Li‐OPA on LPSClBr Electrolytes

**Figure 1:**
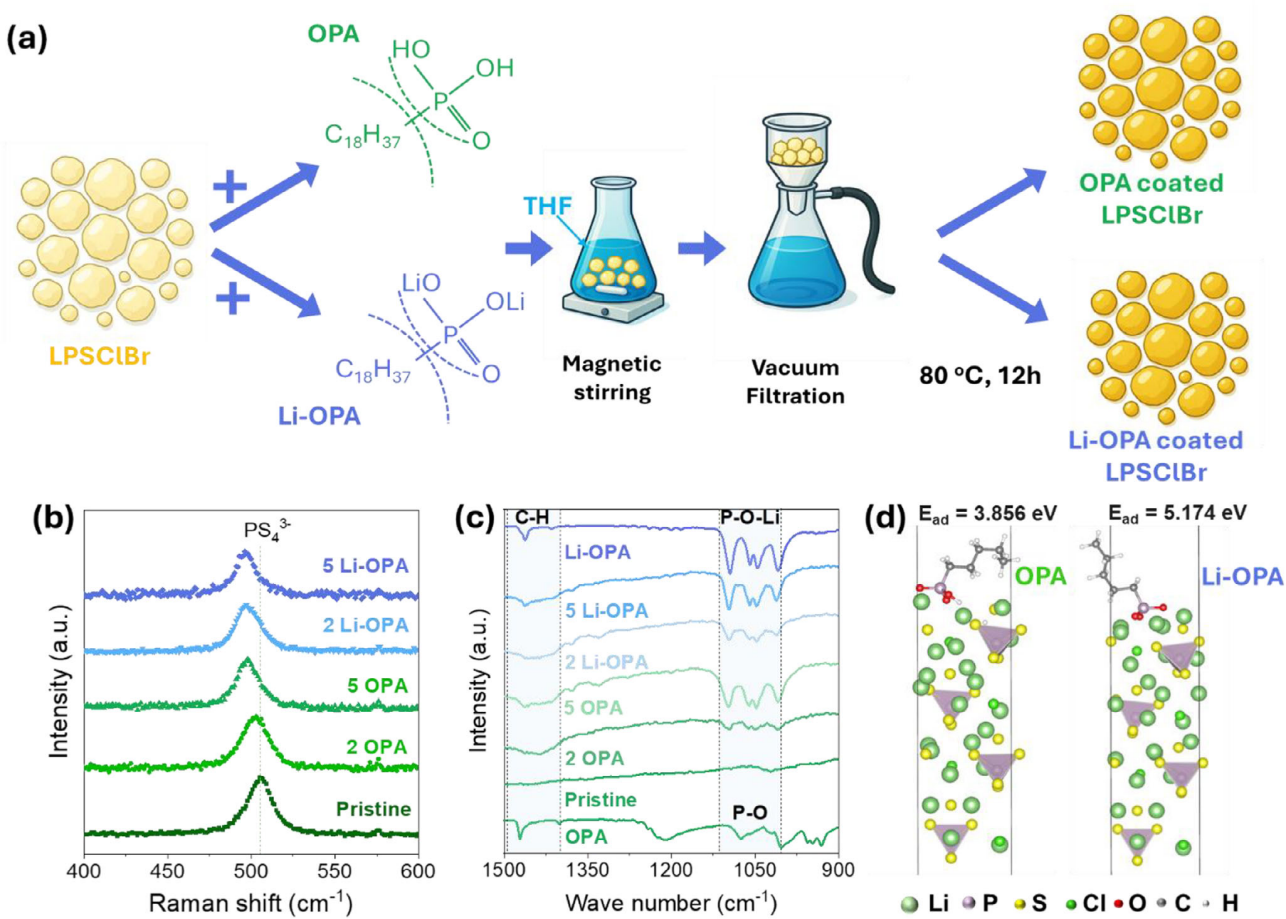
a) Schematic illustration of the OPA/Li‐OPA surface functionalization process on LPSClBr solid electrolyte. b) Raman and c) FTIR spectra of pristine LPSClBr, OPA‐coated, and Li‐OPA‐coated LPSClBr with varying additive concentrations, the numbers 2 and 5 represent the 2 and 5 wt% of OPA. d) Atomistic simulation of OPA and Li‐OPA adsorption on the LPSCl surface, showing the optimized geometry obtained via DFT calculations.

Li‐OPA was prepared through a simple acid–base neutralization reaction between OPA and lithium hydroxide. Fourier‐transform infrared spectroscopy (FTIR) analysis was performed (Figure S1a, Supporting Information) on OPA and its lithiated form. The characteristic P–O stretching vibrations of OPA appear at 1200, 1075, and 990 cm^−1^, corresponding to the phosphonic acid functional groups. Upon lithiation, these peaks shift to 1100, 1050, 1045, and 1000 cm^−1^ indicating the formation of P─O─Li bonds. These spectral shifts are consistent with previous studies, in which pH‐driven modification of OPA resulted in similar vibrational changes.^[^ ^44^ ^]^ The coated electrolytes were prepared via a wet‐mixing approach, as illustrated in **Figure** **1**a, by blending given amounts of either OPA or Li‐OPA with LPSClBr (see Methods Section for details). While “2 OPA” denotes a composition containing 2 wt.% OPA and 98 wt.% LPSClBr, and “2 Li‐OPA” refers to 2 wt.% Li‐OPA with 98 wt.% LPSClBr. The chemical interactions among pristine LPSClBr, OPA‐coated LPSClBr, and Li‐OPA‐coated LPSClBr were systematically investigated using Raman and FTIR spectroscopy analyses. Figure 1b shows a redshift in the PS_4_^3−^ stretching mode (505 cm^−1^) for all coated samples, indicating a perturbation in the P─S bonding environment, likely due to noncovalent interactions at the interface between the electrolyte and the additive. The Li‐OPA‐coated sample exhibited a more pronounced redshift to 495 cm^−1^, suggesting stronger surface interactions. Similarly, FTIR analysis (also see Figure S1b, Supporting Information) revealed a redshift of 5 cm^−1^ in the PS_4_^3−^ stretching mode at 580 cm^−1^ in OPA‐coated samples, along with peak broadening and intensity reduction, indicative of local disorder and Li extraction. These vibrational changes point to Lewis acid–base interactions between the phosphonic acid headgroups and the P–S framework of LPSClBr. Notably, characteristic vibrational modes of Li‐OPA were observed in both OPA‐ and Li‐OPA‐coated samples (Figure 1c), suggesting that OPA undergoes in situ lithiation at the interface via Li‐ion migration from LPSClBr. Additionally, the FTIR spectrum also revealed (Figure S1c, Supporting Information) C─H stretching vibration of the octyl moiety between 2800 and 3000 cm^−1^, indicating successful surface functionalization.

To support these experimental findings, atomistic simulations were performed using the point edge transformer (PET) architecture and trained on the Massive Atomistic Diversity (MAD) universal potential to investigate the interaction between OPA and the LPSCl surface. For computational efficiency, a simplified model was adopted: LPSCl was used as a representative surface for LPSClBr, and the OPA molecule was modelled with a five‐carbon alkyl chain instead of the full 18‐carbon chain used in experiments. This simplification is justified by Monte Carlo simulations of the LPSClBr (100) surface (Figure S2, Supporting Information), which show that even when Br atoms are initially placed at the surface, the energetically preferred configurations feature Cl atoms at the surface while Br migrates into the bulk. This indicates that the surface chemistry of LPSClBr is dominated by Cl termination, making LPSCl an appropriate surrogate for modelling surface interactions. This reduction in chain length preserves the essential interfacial chemistry of the phosphonic acid headgroup while significantly lowering computational cost. Across multiple initial configurations, the phosphonic acid headgroup is consistently oriented toward and coordinated with Li sites on the LPSCl surface (Figure 1d), indicating strong surface affinity and a thermodynamically favorable adsorption geometry. Density functional theory (DFT) calculations were used (Figure 1d) to quantify the adsorption strength of both protonated OPA (OPA‐H) and its lithiated form, Li‐OPA, on the LPSCl surface. The results revealed higher adsorption energy for Li‐OPA (5.174 eV) compared to OPA‐H (3.856 eV), indicating stronger thermodynamic driving forces for interfacial anchoring in the lithiated case. These findings correlate well with the larger spectral shifts observed experimentally (see Figure 1b; Figure S1c, Supporting Information) and support the hypothesis that lithiation enhances molecular binding and interfacial integration. Further insight into the lithiation mechanism was provided by a supplementary molecular dynamics video (see Video S1a, Supporting Information), which shows proton transfer from OPA to sulfur atoms on LPSClBr and subsequent migration of Li ions to the phosphonic acid oxygen, confirming in situ lithiation upon coating. In contrast, Video S1b (Supporting Information) demonstrates that pre‐lithiated Li‐OPA interacts more strongly and closely with the surface, consistent with the higher adsorption energy calculated by DFT.

X‐ray photoelectron spectroscopy (XPS) provided additional evidence of surface modification (Figure S3a, Supporting Information). In both 2 wt.% OPA‐ and 2 wt.% Li‐OPA‐coated LPSClBr, the S 2p peak exhibited only a negligible shift of 0.1 eV compared to pristine LPSClBr (161.6 eV), suggesting that the LPSClBr framework remained chemically intact and that the coating process did not induce significant degradation. The preservation of the PS_4_^3−^ signal intensity further supports the structural stability of the underlying electrolyte. Moreover, the P 2p spectra revealed the presence of P–O species near 133 eV, consistent with the phosphonic acid functional groups in OPA and Li‐OPA, confirming the formation of a phosphate‐rich interfacial layer. These phosphate signals are attributed to the organic coating on the surface rather than the decomposition of the electrolyte. These observations were further supported by X‐ray diffraction (XRD) analysis (Figure S3c, Supporting Information). All coated samples retained diffraction patterns nearly identical to the pristine LPSClBr, with no significant peak shifts or broadening, indicating that the crystalline structure of the electrolyte was preserved following surface treatment. This confirms that both OPA and Li‐OPA coatings are structurally non‐destructive and that the functionalization occurs primarily at the surface without compromising the bulk crystallinity. Transmission Electron Microscopy (TEM) and XPS depth profiling were performed to evaluate the coverage and uniformity of the coating on LPSClBr (Figure S4a–d, Supporting Information). TEM images of pristine LPSClBr particles (Figure S4a, Supporting Information) show a crystalline interior without an observable amorphous surface region, providing a baseline for comparison with coated samples. In contrast, TEM images of Li‐OPA–coated LPSClBr particles (Figure S4c, Supporting Information) reveal an amorphous region at the particle surface, consistent with the presence of a surface‐modified layer. Fast Fourier Transform (FFT) patterns of the coated sample confirm that the particle interior remains crystalline while the outer region is amorphous. However, given the beam sensitivity of sulfide electrolytes and organic species, the apparent morphology and thickness of this amorphous layer may be influenced by electron‐beam exposure. To complement the TEM observations, XPS depth profiling was conducted on both pristine and coated samples. The depth profiles reveal that the Li‐OPA–coated LPSClBr (Figure S4d, Supporting Information) consistently exhibits higher concentrations of C 1s, O 1s, P 2p, and Li 1s across multiple sputtering intervals compared to the pristine material. As the sputtering‐rate calibration for LPSClBr is not available, the profiles are interpreted qualitatively. Together, the TEM and XPS results suggest the formation of a chemically distinct surface region upon Li‐OPA treatment.

### 2.2 Stability and Electrochemical Behavior of Modified LPSClBr Electrolytes

Electrochemical impedance spectroscopy (EIS) was used to measure the Li‐ion conductivity of pristine LPSClBr, OPA‐coated LPSClBr, and Li‐OPA‐coated LPSClBr (Figure S5a–c, Supporting Information). The pristine sample exhibited the expected high ionic conductivity of 5.7 mS cm^−1^, characteristic of a well‐structured argyrodite framework with abundant mobile Li ions. In contrast, the 5 wt.% OPA‐coated sample displayed a significant reduction in ionic conductivity, reaching only 0.8 mS cm^−1^. This decrease aligns with the FTIR observations of PS_4_^3−^ redshifts and intensity losses, supporting the conclusion that Li extraction by OPA during surface modification depletes mobile Li carriers. In contrast, the Li‐OPA‐coated LPSClBr exhibited ionic conductivity of 2.4 mS cm^−1^. Although this value remains lower than that of pristine LPSClBr, it is markedly higher than that of OPA‐coated samples. This enhancement can be attributed to the Li‐rich nature of Li‐OPA, which helps replenish Li vacancies at the interface, as supported by DFT calculations and Raman shifts indicating stronger interfacial interactions. Additionally, the chemically stable interface formed by Li‐OPA mitigates structural damage and facilitates Li‐ion conduction.

**Figure 2:**
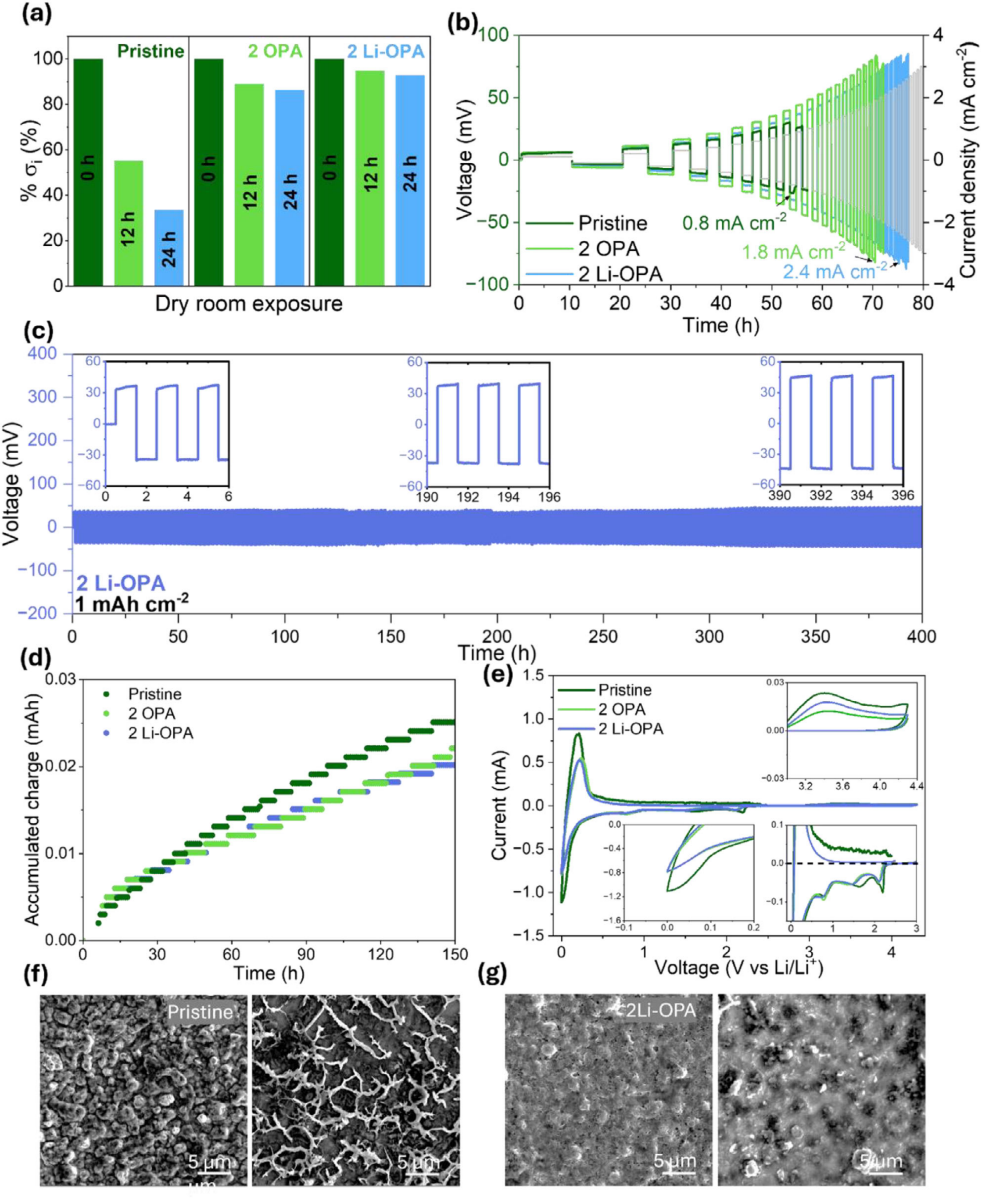
a) Li‐ion conductivity of pristine LPSClBr and modified LPSClBr before and after 12 h and 24 h dry room (dew point −50 °C) exposure. b) Critical current density of Li symmetrical cells containing pristine LPSClBr and modified LPSClBr with a constant capacity of 1.0 mAh cm^−2^. c) Symmetrical cell performance of Li|2 Li‐OPA|Li at 1.0 mA cm^−2^, 1.0 mAh cm^−2^. d) The accumulated charge from CTTA measurements as a function of time, in a cell configuration of stainless‐steel foil|LPSClBr‐coated LPSClBr|Li. e) Cyclic voltammetry profile of 1^st^ cycle for Li|Pristine LPSClBr, 2 OPA‐coated LPSClBr, and 2 Li‐OPA‐coated LPSClBr|VGCF cells. SEM images of stainless‐steel current collectors after 15 h CTTA measurement of f) Pristine and g) 2 Li‐OPA coated LPSClBr.

The stability of pristine and coated LPSClBr under dry‐room conditions (dew point −50 °C) was evaluated by monitoring the retention of ionic conductivity after 12 and 24 h of exposure. As shown in **Figure** **2**a, the pristine LPSClBr exhibited a pronounced decline in ionic conductivity, retaining only 55% after 12 h and dropping further to 30% after 24 h, suggesting a chemical decomposition of LPSClBr upon reacting with moisture. In contrast, 2 wt.% OPA‐coated sample retained 87% of its initial conductivity after 12 h and 84% after 24 h, demonstrating enhanced resistance to degradation. Notably, 2 wt.% Li‐OPA‐coated LPSClBr maintained over 92% of its original conductivity after 24 h exposure, indicating superior interfacial and structural stability under dry room conditions. Post‐mortem XRD analysis was conducted on pristine and Li‐OPA–coated LPSClBr samples after exposure to controlled dry‐room environments with dew points of −50 °C (0.1% RH, 25 °C) for 12 h and −10.1 °C (9% RH, 25 °C) for 4 h. As shown in Figure S6a (Supporting Information), the pristine electrolyte exhibited rapid decomposition under −50 °C dew‐point conditions, as evidenced by the appearance of new diffraction peaks corresponding to secondary phases. In contrast, the coated sample maintained its original diffraction pattern without noticeable phase changes or additional reflections, indicating enhanced resistance to moisture‐induced degradation. After exposure to the −10.1 °C dew‐point environment for 4 h (Figure S6c, Supporting Information), the pristine LPSClBr was almost completely degraded, with the characteristic reflections of LPSClBr nearly disappearing, signifying extensive structural breakdown. Meanwhile, the Li‐OPA–coated sample retained the primary diffraction peaks along with the appearance of additional reflections indicating the formation of a minor amount of decomposition products. FTIR spectra (see Figure S6b,d, Supporting Information) corroborate these findings, showing that both samples experienced broadening of the characteristic PS_4_^3−^ vibrational modes, consistent with breakdown of the thiophosphate framework. In the pristie sample, new absorptions emerged at 780 cm^−1^, assignable to P─O─P stretching of polyphosphate species, at 1051 cm^−1^, corresponding to asymmetric P─O vibrations of Li_3_PO_4_, and at 885 cm^−1^, attributable to Li_2_CO_3_ and OH bonds appear as a strong, broad absorption band in the 2500–3600 cm^−1^ region of the spectrum formed via reaction with humid. These new features were substantially less pronounced in the Li‐OPA–coated sample, which showed reduced peak broadening and fewer secondary absorptions, indicating that while the coating does not fully suppress degradation, it significantly retards the hydrolysis and oxidation pathways that lead to phosphate and carbonate formation. These results highlight the protective effect of organic surface functionalization, with the hydrophobic alkyl chains in OPA and the Li‐rich interface in Li‐OPA to effectively suppress conductivity loss even under prolonged dry room exposure. Electronic conductivity, measured using chronoamperometry, further confirmed the insulating nature of both coatings (see Figure S5 g, Supporting Information). The pristine LPSClBr exhibited an electronic conductivity of 10^−9^ S cm^−1^, while both 2 wt.% OPA and 2 wt.% Li‐OPA samples showed reduced values of 5 × 10^−10^ S cm^−1^. This suppression is attributed to the formation of electronically insulating interfacial layers. The alkyl chains in OPA provide passive insulation, whereas the lithiated structure of Li‐OPA additionally stabilizes the interfacial chemistry and supports selective Li‐ion transport, as evidenced by reduced parasitic reactions and preserved solid electrolyte interphase (SEI) structure in the subsequent analyses.

Critical current density (CCD) measurements at an areal capacity of 1 mAh cm^−2^ (see Figure 2b; Figures S7 and S8, Supporting Information) further revealed the effect of surface modifications. The pristine LPSClBr exhibited a CCD of only 0.8 mA cm^−2^ before short‐circuiting, while 2 wt.% OPA and Li‐OPA coatings significantly enhanced CCD values to 1.8 and 2.4 mA cm^−2^, respectively. The improvement with Li‐OPA is consistent with stronger molecular binding, as supported by the DFT calculations, and its stabilizing effect at the Li | electrolyte interface. However, increasing the coating to 5 wt.% led to decreased CCD values, likely due to excessive surface passivation and increased interfacial resistance. Additionally, the voltage profiles further corroborate these results; the pristine LPSClBr showed the lowest initial overpotential, consistent with its high intrinsic ionic conductivity, but it failed rapidly due to interfacial degradation. In contrast, both OPA‐ and Li‐OPA‐coated samples exhibited slightly higher overpotentials yet demonstrated markedly improved cycling stability by mitigating parasitic reactions at the interface and suppressing Li dendrite growth.

Long‐term Li plating and stripping behaviour in symmetric cells (see Figure 2c; Figure S9, Supporting Information) further emphasized the superior interfacial stability provided by Li‐OPA coatings. The pristine LPSClBr cell short‐circuited during the first cycle due to uncontrolled dendritic growth and rapid interfacial breakdown. In contrast, the 2 wt.% OPA‐coated sample achieved extended cycling of ≈150 h, while the 2 wt.% Li‐OPA‐coated sample maintained stable voltage profiles with minimal polarization for over 400 h at a current density of 1.0 mA cm^−2^ (1.0 mAh cm^−2^), demonstrating excellent dendrite suppression and a robust interphase formation. To support these observations, area‐specific resistance (ASR) was extracted from the voltage profiles (see Figure S10, Supporting Information). Although the pristine sample initially showed the lowest ASR, it underwent a sharp drop in resistance upon short‐circuiting, clearly visible in the ASR plots. Conversely, the Li‐OPA‐coated sample exhibited stable ASR over prolonged cycling, highlighting the electrochemical and mechanical stability of the interfacial layer. Coulometric Titration Time Analysis (CTTA)^[^ ^45^ ^]^ (see Figure 2d; Figure S11, Supporting Information) further confirmed the suppression of parasitic side reactions. The pristine sample exhibited a cumulative charge q_Σ_ = Σq(τ_i_) = 28 µAh, corresponding to q_A,Σ_ = 44 µAh cm^−2^ after 150 h and 25 deposition cycles, indicating continuous Li consumption. Both coatings significantly reduced accumulated charge by 10 µAh cm^−2^, reflecting the protective effect of the coatings in minimizing interfacial decomposition. Cyclic voltammetry analysis (see Figure 2e) was performed to further support these findings. Pristine LPSClBr exhibited high current densities and severe voltage hysteresis, indicative of unstable Li plating/stripping. In contrast, coated samples showed reduced current density, lower hysteresis, and well‐defined redox peaks. Scanning electron microscopy images obtained (see Figures 2f,g) after 150 h of CTTA measurement revealed distinct SEI morphologies. The pristine sample formed a porous, irregular SEI with poor interfacial contact, while the Li‐OPA‐coated sample exhibited a dense, uniform interphase. This morphological difference is attributed to two synergistic effects of the Li‐OPA coating: i) the organic layer serves as a physical barrier limiting direct contact between the electrolyte and reactive surfaces such as Li metal and the current collector, and ii) the phosphonic acid headgroups interact non‐covalently with PS_4_^3−^ units in the LPSClBr matrix, helping to stabilize the polyanionic framework and suppress interfacial decomposition. Together, these effects result in a more controlled interfacial chemistry and enhanced long‐term electrochemical stability.

### 2.3 Full‐Cell Performance in Solid‐State Batteries

**Figure 3:**
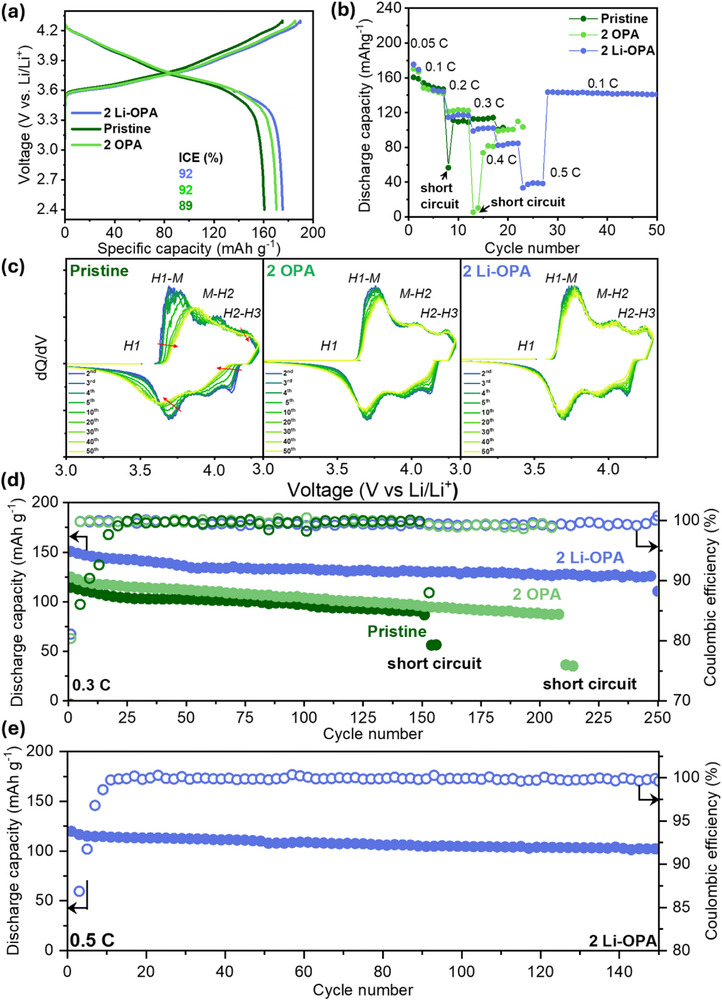
ASSB performance for 50 µm Li|Pristine or coated LPSClBr|NCM811 composite cells with an active material loading of 9.1 mg cm^−2^. a) First‐cycle charge and discharge profiles at 0.05C. b) Rate performance across various current densities. c) Differential capacity plots of full cells containing pristine LPSClBr, 2 OPA‐LPSClBr, and 2 Li‐OPA‐LPSClBr. d) Cycling performance at 0.3C e) Cycling performance of 2 Li‐OPA cells at 0.5C.

The electrochemical performance of pristine NMC811 and pristine or coated LPSClBr in ASSBs was systematically evaluated (**Figure** **3**). The first‐cycle charge and discharge profiles (see Figure 3a) reveal a substantial improvement in CE with OPA and Li‐OPA surface coatings. The pristine LPSClBr cell exhibits a CE of only 89%, indicative of significant irreversible capacity loss. In contrast, the 2 wt.% OPA‐ and Li‐OPA‐coated LPSClBr‐based SSBs achieved improved CEs of 92% underscoring the role of surface functionalization in mitigating interfacial degradation.

Figure 3b illustrates the rate capability of the different SSBs. The pristine cell suffers severe performance degradation and fails at a C‐rate of 0.2C. The 2 OPA‐coated cell extends operational stability to 0.3C, while the 2 wt.% Li‐OPA‐coated cell maintains stable cycling even at 0.5C and recovers its initial capacity upon returning to 0.1C, indicating superior interfacial stability and mechanical resilience at higher current densities. The interfacial compatibility between the solid electrolytes and the NMC811 cathode was assessed by differential capacity (dQ/dV) analysis over the 1^st^ to 50^th^ cycles at 0.1 C (Figure 3c). For the cell employing pristine LPSClBr, the dQ/dV profiles display a pronounced and progressive attenuation of peak intensity with increasing cycle number, indicating the degradation of redox activity over time. Notably, the H1–M redox transition peak undergoes a significant voltage shift of ≈0.16 V toward higher potentials after 50 cycles, reflecting increased overpotential and interfacial polarization. This behavior suggests a substantial loss of electrochemical and structural reversibility at the cathode–electrolyte interface.^[^ ^46^ ^]^ Such changes are typically associated with pronounced phase transitions and mechanical degradation in the NMC811 lattice, driven by poor interfacial stability and parasitic side reactions. These processes hinder the reversible diffusion of Li ions and accelerate capacity fading during prolonged cycling.^[^ ^46^ ^]^ In contrast, the 2 wt.% OPA‐coated SSB displays a dQ/dV profile with minimal shifts in both H and H1–M phase transition peaks, indicating improved structural reversibility and suppressed degradation at the interface. Notably, the 2 wt.% Li‐OPA‐coated SSB exhibits the most stable dQ/dV behavior, with defined and well‐preserved H1 and H1–M peaks that remain nearly unchanged over repeated cycling. This enhanced electrochemical stability can be attributed to the more robust and chemically integrated interphase formed by Li‐OPA, as supported by FTIR, Raman, and DFT analyses. The consistent redox behavior reinforces the conclusion that Li‐OPA modification effectively mitigates interfacial decomposition, maintains lattice integrity, and promotes long‐term cathode reversibility in sulfide electrolyte‐based SSBs. Cycling performance at 0.3C (see Figure 3d) further supported these results. After 100 cycles, the pristine LPSClBr, 2 wt.% OPA‐, and 2 wt.% Li‐OPA‐LPSClBr‐based SSBs retained 83.9%, 83.6%, and 85.2% of their initial capacities, respectively. The Li‐OPA‐modified LPSClBr‐based SSB sustained performance beyond 250 cycles at 0.3C, unlike the more pronounced degradation observed in the other cells. Figure 3e demonstrates that the Li‐OPA‐coated sample also maintained a discharge capacity of 90 mAh g^−1^ and 81% retention after 150 cycles at 0.5C, confirming its long‐term stability under demanding conditions.

To further explore cathode–electrolyte interactions in LPSClBr‐based ASSBs, we also investigated LiNbO_3_ (LNO)‐coated NCM811 cathodes paired with either pristine LPSClBr or Li‐OPA‐coated‐LPSClBr (see Figure S12, Supporting Information). Across all electrolyte conditions, cells employing LNO‐coated NCM811 consistently exhibited higher discharge capacities at 0.05 C and improved initial CEs compared to those with uncoated cathodes. The enhanced performance observed in LNO‐coated NCM811‐based cells can be attributed to improved cathode surface characteristics, particularly enhanced Li‐ion transport kinetics. In contrast, the uncoated NCM811 surface is prone to the accumulation of surface impurities such as Li_2_CO_3_ and LiOH during storage, primarily due to their sensitivity to moisture and CO_2_ exposure. These surface contaminants can subsequently impede Li‐ion mobility and interfacial kinetics.^[^ ^46^ ^]^ Notably, the pristine LPSClBr electrolyte combined with LNO‐coated NCM811 shows improved initial capacity and can sustain approximately 20 cycles at 0.1C even after high‐rate cycling at 0.5C. However, while LNO coatings help mitigate interfacial degradation, they are prone to cracking during cycling due to volume changes in cathode particles, leading to coating detachment, increased interfacial resistance, and long‐term performance degradation.^[^ ^10^ ^]^ In contrast, the Li‐OPA coating on LPSClBr provides a more robust and chemically stable interface. When pairing LNO‐coated NCM811 with Li‐OPA‐modified LPSClBr (see Figure S12d, Supporting Information), the cells demonstrate improved capacity retention and sustained cycling performance even after high‐rate operation, indicating the possibility of combining cathode and electrolyte surface engineering to mitigate interfacial degradation and stabilize the electrochemical performance of ASSBs.

### 2.4 Zero‐Excess Li Anode All‐Solid‐State Battery

**Figure 4:**
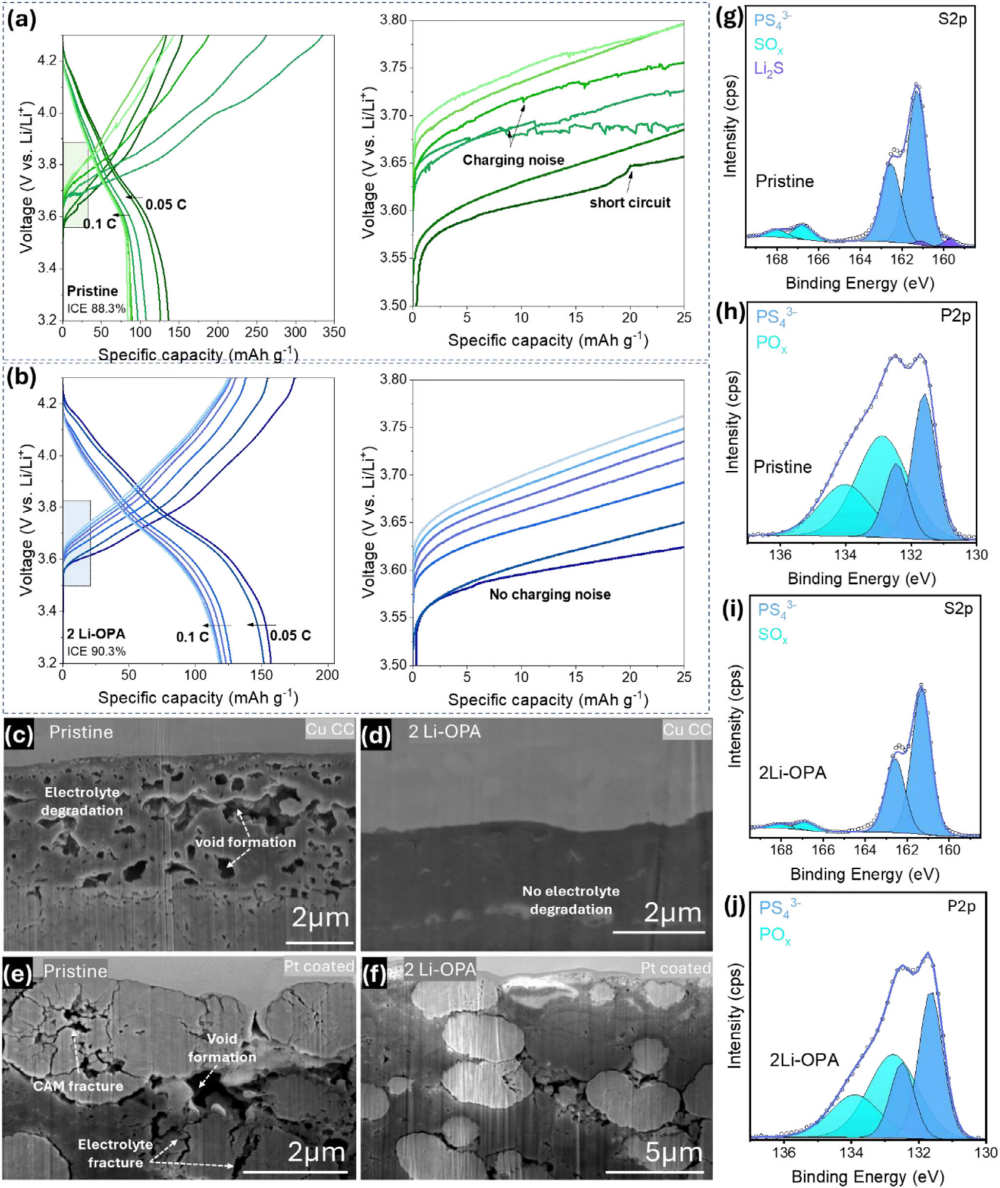
Charge–discharge curves of anode‐free SSBs using a) pristine LPSClBr and b) 2 wt.% Li‐OPA‐coated LPSClBr. Cross‐sectional FIB‐SEM images of the anode side c,d) and cathode–electrolyte interface e,f) reveal improved interfacial contact with 2 Li‐OPA. Post‐cycling XPS g–j) confirms reduced LPSClBr decomposition with 2 Li‐OPA compared to the pristine electrolyte.

The superior electrochemical performance of 2 wt.% Li‐OPA‐LPSClBr also indicates its potential applicability in anode‐less solid‐state battery, which relies heavily on stable Li deposition and minimal interfacial resistance. The key challenge in such systems is maintaining uniform Li plating/stripping without dendrite formation or excessive polarization. The performance of pristine LPSClBr and 2 wt.% Li‐OPA‐coated LPSClBr under anode‐free conditions was evaluated using 12 µm copper foil as the current collector (CC). As shown in **Figure** **4**a, the pristine LPSClBr‐based cell short‐circuited during the first cycle at 0.05C and displayed rapid capacity fading, which can be attributed to parasitic side reactions at the Cu | electrolyte interface. The pristine cell also delivered a relatively low initial Coulombic efficiency (CE) of 88.3%, reflecting pronounced interfacial instability and irreversible capacity loss. When cycled at 0.1C, the pristine cell exhibited extended charging times along with a noisy voltage profile, further confirming poor electrochemical stability. In contrast, the 2 wt.% Li‐OPA‐based SSB (see Figure 4b) achieved a higher initial CE of 90.3% and sustained stable cycling at 0.1C, indicating that the Li‐OPA surface functionalization effectively improved interfacial compatibility, reduced parasitic reactions, and suppressed cell short‐circuiting. To further validate this stability, we conducted Li–Cu asymmetric cell measurements, including stripping/plating stability tests and Aurbach‐type Coulombic efficiency analysis (see Figure S13a, Supporting Information). The Li‐OPA coated LPSClBr exhibits stable Li plating/stripping over 50 h, whereas the cells with the pristine electrolyte experienced premature short‐circuiting, highlighting the insufficient interfacial stability in the absence of surface modification. The CE of the Li‐OPA modified electrolyte is higher than that of the pristine sample and gradually increases with cycling time (See Figure S13b,c, Supporting Information). This increase arises from the progressive formation of a stable passivation layer at the Li|electrolyte interface, which suppresses continuous side reactions and promotes more uniform Li deposition/stripping.^[^ ^47^ ^]^ Aurbach tests at 0.3 mA cm^−2^ (0.3 mAh cm^−2^) and 1 mA cm^−2^ (1 mAh cm^−2^) (Figure S13d,e, Supporting Information) further confirm these findings, where the modified electrolyte achieves high efficiencies of 97.8% and 95.5%, in sharp contrast to the negligible reversibility observed with pristine LPSClBr. While these CE values are lower than the 99.5% typically reported for liquid electrolytes, they are considered excellent in the context of solid‐state batteries. In fact, studies reporting Aurbach‐type CE measurements in sulfide solid electrolytes are extremely scarce; to the best of our knowledge, Ren et al. recently reported an average CE of 95.5% for LPSCl under the condition of 0.1 mA cm^−2^ (0.1 mAh cm^−2^).^[^ ^48^ ^]^ This enhanced performance is attributed to the Li‐OPA layer, which mitigates direct contact between the copper substrate and the reactive LPSClBr, suppresses Li consumption, and promotes uniform Li deposition. To assess morphological changes post‐cycling, cross‐sectional focused ion beam scanning electron microscopy (FIB‐SEM) was performed. In the pristine sample (see Figure 4c), interfacial contact loss and void formation were observed at the Cu | electrolyte interface, indicating severe interfacial degradation. In contrast, the 2 wt.% Li‐OPA‐ LPSClBr‐based cell (also see Figure 4d) retained a robust and intact interface without void formations, indicating improved mechanical and electrochemical stability on the anode side.

Beyond the anode interface, the cathode–electrolyte interface is equally critical for the performance of anode‐free solid‐state batteries. Post‐cycling FIB‐SEM analysis of the pristine LPSClBr cell (Figure 4e) revealed extensive particle fracturing and void formation within the cathode–electrolyte composite, indicating severe interfacial degradation. In contrast, the 2 wt.% Li‐OPA‐based cell (Figure 4f) preserved a denser and more continuous microstructure, with improved particle–electrolyte adhesion and fewer interfacial voids. While the Li‐OPA coating does not completely prevent cathode active material cracking, it effectively stabilizes the electrolyte–cathode contact and mitigates the propagation of interfacial degradation, thereby enhancing long‐term electrochemical performance. To further investigate chemical stability, post‐cycling XPS analysis was also conducted on the pellet surface of the cathode composites from the anode‐free SSBs (see Figures 4g–j) after two cycles at 0.05C. In the pristine sample, S 2p spectra revealed the formation of Li_2_S and an increased concentration of oxidized sulfur species (SO*_x_*), both of which signify extensive decomposition of the sulfide electrolyte. Li_2_S, which forms as a degradation product of LPSCl, is generally considered an unfavorable component of the SEI because of its poor electronic conductivity.^[^ ^49^, ^50^ ^]^ Furthermore, the phosphate (PO*_x_*) on the P2p spectra in the pristine sample shows significantly higher intensity compared to the 2 wt.% Li‐OPA‐coated LPSClBr. A portion of the PO*_x_* species in 2 wt.% Li‐OPA‐LPSClBr can be attributed to the pre‐existing phosphonic acid layer introduced during the surface modification process. The comparatively lower formation of additional PO*_x_* species in the coated LPSClBr reflects suppressed interfacial decomposition and highlights the protective role of the Li‐OPA coating in stabilizing the electrolyte–electrode interface. These findings suggest that Li‐OPA‐coated LPSClBr could serve as a promising electrolyte candidate for next‐generation anode‐free SSBs, offering improved cycling stability, lower interfacial resistance, and enhanced Li reversibility.

## 3 Conclusion

This work presents a scalable, single‐step, electrolyte‐centered organic surface functionalization strategy to stabilize interfaces of argyrodite‐type solid electrolytes in all‐solid‐state batteries. By coating LPSClBr with lithiated octadecyl phosphonic acid, Li‐OPA, the interfacial degradation at both anode and cathode interfaces is simultaneously mitigated. Li‐OPA forms a chemically and electrochemically stable interphase without compromising structural integrity or ionic transport in LPSClBr. The Li‐OPA coating significantly enhances interfacial stability, moisture resistance under dry room conditions, and electrochemical performance of LPSClBr, achieving high critical current densities, extended cycling life, and improved Coulombic efficiency in both excess and anode‐less SSB cell configurations. These results demonstrate the potential of Li‐OPA as an organic interface modifier for next‐generation ASSBs based on LPSClBr solid electrolyte with Li metal anodes and high‐voltage cathodes.

## 4 Experimental Section

### Materials

The argyrodite Li_6_PS_5_Cl_0.5_Br_0.5_, with particle sizes of 2 µm for the solid electrolyte layer and 0.7 µm for the composite cathode, was synthesized following previously reported methods.^[^ ^51^ ^]^ Octadecyl phosphonic acid (OPA, >97%, Apollo Scientific) and lithium hydroxide (LiOH, 98%, Sigma Aldrich) were dried at 90 °C under vacuum for 12 h to eliminate moisture. Anhydrous tetrahydrofuran (THF) stabilized with molecular sieves was purchased from Sigma Aldrich. NMC811 and LiNbO_3_‐coated NMC811 cathode powder (1 wt.% LiNbO_3_ coating) was sourced from MSE Supplies. Vapor‐grown carbon fibers (VGCF) were obtained from SHOWA DENKO. Lithium foil (50 µm thick) was supplied by China Energy Lithium Co., Ltd. Nickel foil was procured from Guangdong Canrd New Energy Technology Co., Ltd., China.

### Preparation of Pre‐Lithiated OPA

Prelithiated OPA (Li‐OPA) was synthesized by reacting 1.0 g of octadecyl phosphonic acid (2.99 mmol, 1.0 eq) with 0.143 g of dry lithium hydroxide (5.98 mmol, 2.0 eq) in 100 mL of THF. The reaction mixture was stirred continuously at 40 °C for 24 h to ensure complete lithiation. Afterward, the solvent was evaporated under reduced pressure at 50 °C and 170 mbar, yielding a solid powder. This powder was further dried at 80 °C for 12 h to remove any residual solvent and give the final product a 78% yield.

### Surface Modification of LPSClBr

LPSClBr mixed with OPA or Li‐OPA additives was prepared by blending the stoichiometric amounts of each additive with LPSClBr (e.g., ′2 OPA′ indicates 2 wt.% OPA and 98 wt.% LPSClBr, and ′2 Li‐OPA′ indicates 2 wt.% Li‐OPA and 98 wt.% LPSClBr). The mixture was suspended in 5 mL of anhydrous tetrahydrofuran (THF) and stirred for 10 min to achieve uniform dispersion. Following stirring, the suspension was vacuum‐filtered and rinsed three times with fresh THF to remove any unreacted residues. The resulting solid powder was collected and dried at 80 °C for 12 h to ensure complete removal of residual solvent and stabilize the composite material.

### X‐ray Diffraction (XRD)

XRD patterns were acquired using a Bruker D8 Advance diffractometer operating in reflection mode with Cu Kα_1_ and Kα_2_ radiation. The measurements were performed over a 2θ range of 2°–90° with a step size of 0.01° and a step time of 0.05 s. To prevent air exposure, all samples were sealed in custom‐made air‐tight sample holders covered with polyethylene foil during data collection.

### Fourier Transmission Infrared Spectroscopy (FTIR)

FTIR spectra were collected using a PerkinElmer Frontier spectrometer equipped with a PIKE GladiATR module. The measurements were conducted over the spectral range of 4000–250 cm^−1^.

### Raman Spectroscopy

Raman imaging was carried out using a WITec Alpha300R Raman confocal microscope (WITec, Ulm, Germany), equipped with a Zeiss 10× or 20× objective lens. A 532 nm diode laser with a maximum output power of 125 mW was fiber‐coupled to the microscope for excitation. To maintain sample integrity and prevent environmental contamination, air‐tight sample holders covered with polyethylene foil were used throughout the measurements.

### Scanning Electron Microscopy (SEM) and Cross‐Sectional Analysis

Surface morphology and cross‐sectional structures of pristine and coated LPSClBr electrodes were examined using a Thermo Fisher Scientific Scios 2 focused ion beam scanning electron microscope (FIB‐SEM). Samples were prepared in an Ar‐filled glovebox and transferred using an airtight holder to minimize air and moisture exposure. For cross‐sectional analysis, a protective platinum layer was first deposited on the electrode surface to prevent ion‐beam–induced damage during milling. Cross‐sections were then prepared by Ga⁺ ion milling under low beam current conditions, followed by high‐resolution SEM imaging to evaluate particle–electrolyte adhesion and the microstructural integrity after cycling. Top‐view SEM imaging was also performed to assess the surface morphology of the cathode–electrolyte composites.

### Transmission Electron Microscopy (TEM)

The morphology and coating structure of the pristine and Li‐OPA–coated LPSClBr particles were examined using a Talos L120C G2 transmission electron microscope (Thermo Fisher Scientific) operated at an accelerating voltage of 120 kV. Samples were prepared inside an Ar‐filled glovebox to minimize exposure to air and moisture. For TEM analysis, the powders were dispersed in anhydrous THF, drop‐cast onto carbon‐coated Cu grids, and sealed in an airtight transfer holder prior to insertion into the microscope. High‐resolution TEM (HRTEM) imaging and Fast Fourier Transform (FFT) analysis of the HRTEM images were performed to evaluate the crystallinity of the underlying LPSClBr.

### Density Functional Theory (DFT)

Atomistic simulations were performed using the PET‐MAD universal potential,^[^ ^52^ ^]^ which is based on the unconstrained point edge transformer (PET) architecture^[^ ^53^ ^]^ and trained on the Massive Atomistic Diversity (MAD) dataset.^[^ ^54^ ^]^ This dataset comprises nearly 100 000 structures spanning 85 elements, computed at the PBEsol level of theory. The PET‐MAD potential enabled fast and efficient exploration of the configurational space of OPA molecules on the LPS surface. To obtain accurate adsorption energies, PET‐MAD relaxed structures were further relaxed using density functional theory (DFT) with the regularized strongly constrained and appropriately normed (rSCAN) meta‐GGA functional with D3 dispersion correction,^[^ ^55^, ^56^ ^]^ as implemented in VASP.^[^ ^57^, ^58^ ^]^ The adsorption energies were then derived from these DFT calculations.

### Moisture Stability of Surface‐Modified LPSClBr

To simulate environmental exposure, 100 mg of pristine, 2 wt.% OPA‐ and 2 wt.% Li‐OPA‐coated LPSClBr powder was evenly spread onto aluminum foil and exposed to a dry room environment with the dew point of −50 °C. The samples were left exposed for 12 and 24 h. Following exposure, the powder was carefully collected and sealed in an air‐tight container to prevent additional environmental contamination before subsequent conductivity measurements. The additional test for humidity stability of pristine and coated LPSClBr electrolytes was evaluated, the samples were placed in a dry room with −50 dew point (0.1% RH, 25 °C) for 12 h and in a sealed chamber with a dew point of −10.1 °C dew point (9% RH, 25 °C) for 4 h. After exposure, the electrolytes were immediately transferred back into an Ar‐filled glovebox, where they were subjected to post‐mortem characterization by XRD and FTIR to assess structural and chemical changes.

### Electrochemical Impedance Spectroscopy (EIS)

To evaluate the ionic conductivity of LPSClBr (pristine, OPA‐coated, and Li‐OPA‐coated), pellets were prepared by pressing 80 mg of LPSClBr powder in a 9 mm polyether ether ketone (PEEK) die (Qingdao Gufeng) at 370 MPa using stainless steel plungers. The resulting pellets, with a thickness of ≈0.8 mm measured using a Vernier calliper, were tested at room temperature using a Biologic VSP‐300 potentiostat, at 20 MPa external pressure. The impedance was measured over a frequency range of 1 Hz to 7.0 MHz with an AC signal amplitude of 10 mV. Nyquist plots were analyzed using Biologic's analysis software to determine the total resistance (R). The ionic conductivity (σ, in mS cm^−1^) was calculated using the Equation (2):

(2) $$\sigma =\frac{d}{R\;\times A}$$

Where, d is pellet thickness (cm), R is the resistance (Ω), and A is the area of the pellet (cm^2^).

### Electronic Conductivity of Surface‐Modified LPSClBr

Electronic conductivity was assessed using DC polarization via the chronoamperometry method. A constant bias potential of 1 V was applied across the LPSClBr pellets for 1800 s using a Biologic VSP‐300 potentiostat. When the voltage is applied, the current initially changes due to capacitive or transient effects but eventually reaches a steady‐state value. This steady‐state current reflects the electronic transport through the material. The electronic conductivity (σ_e_, in S/cm) can then be calculated using the Equation (3):

(3) $$\sigma_{e}=\frac{I_{SS}\;\times \;d}{V\;\times A}$$

Where I_SS_ is the steady‐state current, d is pellet thickness (cm), V is the applied voltage (V), and A is the area of the pellet (cm^2^).

### Symmetrical Cell

Symmetric cells were constructed to investigate the interfacial stability between LPSClBr and the Li metal anode. To achieve this, 80 mg of electrolyte powder was compacted under 3 tons of pressure to form a solid pellet. A thin layer of lithium metal (50 µm on copper foil) was then placed on both sides of the pellet, followed by the application of 0.5 tons of pressure to ensure optimal interfacial contact. The CCD test at 20 MPa external pressure was conducted at a constant capacity of 1 mAh cm^−2^, with the current density increasing from 0.1 to 3.5 mA cm in 0.1 mA cm^−2^ steps. Long‐term lithium plating and stripping cycling was performed at a fixed capacity of 0.1 mAh cm^−2^ with a constant current of 0.1 mA cm^−2^. The ASR was calculated using Equation (4):

(4) $$ASR=\frac{V\;\times A}{2I}$$

Where ASR refers to the area‐specific resistance (Ω·cm^2^). A denotes the electrode surface area (cm^2^), V is the overpotential, and I is the applied current.

### Asymmetrical cell test

Li|Cu foil cells were assembled to evaluate the Coulombic efficiency (CE) of lithium plating and stripping through the LPSClBr electrolyte. In each case, 80 mg of electrolyte powder was compacted under 3 tons of pressure to form a solid pellet, which was then sandwiched between the working electrode (Cu foil) and a lithium counter electrode. The stack was compressed under an external pressure of 20 MPa during cycling. Lithium plating was carried out at a fixed areal capacity of 1.0 mAh cm^−2^, followed by a rest period of 5 min, and subsequent stripping at the same current density until a cutoff of 1.0 V. The Coulombic efficiency (CE) was calculated using Equation (5):

(5) $$CE=\frac{Q_{strip}}{Q_{plate}}$$

where Q_strip_ and Q_plate_ represent the plated and stripped capacities, respectively.

### Aurbach Test in Li|Cu Half‐Cells for CE Calculation

Li|Cu cells were assembled to evaluate the CE of lithium plating and stripping through the LPSClBr electrolyte. In each case, 80 mg of electrolyte powder was compacted under 3 tons of pressure to form a solid pellet, which was then sandwiched between the working electrode (Cu foil) and a thick (600 µm) lithium counter electrode. The stack was compressed under an external pressure of 20 MPa during cycling. For the Aurbach test, a defined lithium reservoir (3 or 10 mAh cm^−2^) was first plated onto the Cu substrate. A smaller portion of this charge (Q_C_ = 0.3 mAh cm^−2^ at 0.3 mA cm^−2^ or 1 mAh cm^−2^ at 1 mA cm^−2^) was then repeatedly cycled for 10 cycles. After completion of cycling, the remaining lithium reservoir was exhaustively stripped at the initial current density (corresponding to Q_T_) until a cut‐off voltage of 1.0 V. The final stripping charge (Q_S_), corresponding to the quantity of lithium remaining after cycling, was measured. The average CE was then calculated using Equation (6):

(6) $$CE_{Average}=\frac{nQ_{C}+Q_{S}}{nQ_{C}+Q_{T}}$$

where n is the number of cycles, Q_T_ is the initial reservoir capacity, Q_C_ is the cycled capacity, and Q_S_ is the final stripping capacity. In this approach, the CE reflects the reversibility of lithium plating/stripping while minimizing artifacts from the Cu substrate.

### Coulometric Titration Time Analysis (CCTA)

CTTA was performed using a Ni disk as the working electrode and lithium metal as both the counter and reference electrodes. In the cell assembly, the Ni disk was positioned at the base of the cell, followed by pressing 80 mg of LPSClBr on top at 3 tons pressure. A lithium foil disc supported on a Cu substrate was then pressed onto the electrolyte layer at 0.5 tons. The fully assembled cell was placed in a pressure frame under 20 MPa and allowed to stabilize for 6 h, during which the cell voltage settled between 2.3 and 2.5 V *vs* Li/Li⁺. The measurements were conducted using a Land 2001A battery system. During each titration step, 1 µAh of lithium was plated with a current of 1 mA over 6 min. After the plating step, the cell was left at open circuit voltage (OCV) until the potential rose above 0.05 V, indicating complete lithium consumption. This titration process was then repeated continuously over 150 h.

### Cyclic Voltammetry (CV)

The electrochemical stability window of the electrolytes was determined using CV. The test cell consisted of LPSClBr pellets, a lithium metal anode on one side, and a composite cathode on the other, containing 10 mg of a 7:3 weight ratio mixture of sulfide electrolyte and vapor‐grown carbon fibers (VGCF). The cathode mixture was homogenized using a Thinky mixer. The CV measurements were performed with a scan rate of 0.1 mV s^−1^ over a voltage range from 0 to 4.3 V vs Li/Li⁺.

### Full‐Cell Assembly

The LiNi_0.8_Co_0.1_Mn_0.1_O_2_ (NCM811) all‐solid‐state full‐cells were assembled using 13.6 mg of NCM811 cathode composite (≈15 mg cm^−2^ loading), 80 mg of LPSClBr electrolyte, and a lithium metal‐foil anode coated on Cu foil. The cathode composite was prepared by mixing NCM811 and LPSClBr in a 70:30 weight ratio using a Thinky mixer for uniform dispersion. LPSClBr powder was first pressed at 3 tons to form a solid electrolyte layer during cell assembly. The cathode composite was evenly distributed on one side of the electrolyte layer and pressed at 3 tons. Finally, a lithium metal‐foil anode coated on Cu foil was placed onto the other side of the solid electrolyte and pressed at 0.5 tons to complete the cell assembly. The fully assembled cell was placed in a pressure frame under 20 MPa and allowed to stabilize for 6 h.

### Full‐Cells with Zero‐Excess Anode

Anode‐free half‐cells were assembled using 80 mg of LPSClBr electrolyte. On one side of the electrolyte layer, 13.6 mg of NCM811 cathode composite (≈15 mg cm^−2^ loading) is pressed, while the other side of the electrolyte layer has a pristine Cu foil as current collector. The fully assembled cell was placed in a pressure frame under 50 MPa and allowed to stabilize for 6 h.

### Cycling Performance Evaluation

The electrochemical performance of the assembled half‐cell was evaluated through rate performance and long‐term cycling tests. The charge–discharge rate was progressively increased from 0.05C for two cycles to 0.1C, 0.2C, 0.3C, 0.4C, and 0.5C, with five cycles performed at each rate. Long‐term cycling stability was evaluated at both 0.3C and 0.5C. All electrochemical tests were conducted under external pressures of 50 MPa.

## Conflict of Interest

The authors declare no conflict of interest.

## Supporting information



Supporting Information

## Data Availability

The data that support the findings of this study are openly available in Zenodo at, https://doi.org/10.5281/zenodo.17816356.
