# Supplementary material for: Molecular Surface Engineering of Sulfide Electrolytes with Enhanced Humidity Tolerance for Robust Lithium Metal All‐Solid‐State Batteries
Source: Adv Mater. 2025 Dec 16;38(9):e15013. doi: 10.1002/adma.202515013 (PMC12902607; doi:10.1002/adma.202515013)
Supplement: Supplementary file 1 — Supporting Information [file ADMA-38-e15013-s001.docx]

Supporting Information

**Molecular Surface Engineering of Sulfide Electrolytes with Enhanced Humidity Tolerance for Robust Lithium Metal All-Solid-State Batteries**

*Laras Fadillah, Leonie Braks, Jihoon Oh, Mingliang Liu, Mounir Mensi, Hanna Türk, Davide Tisi, Michele Ceriotti, Jang Wook Choi*, Ali Coskun**


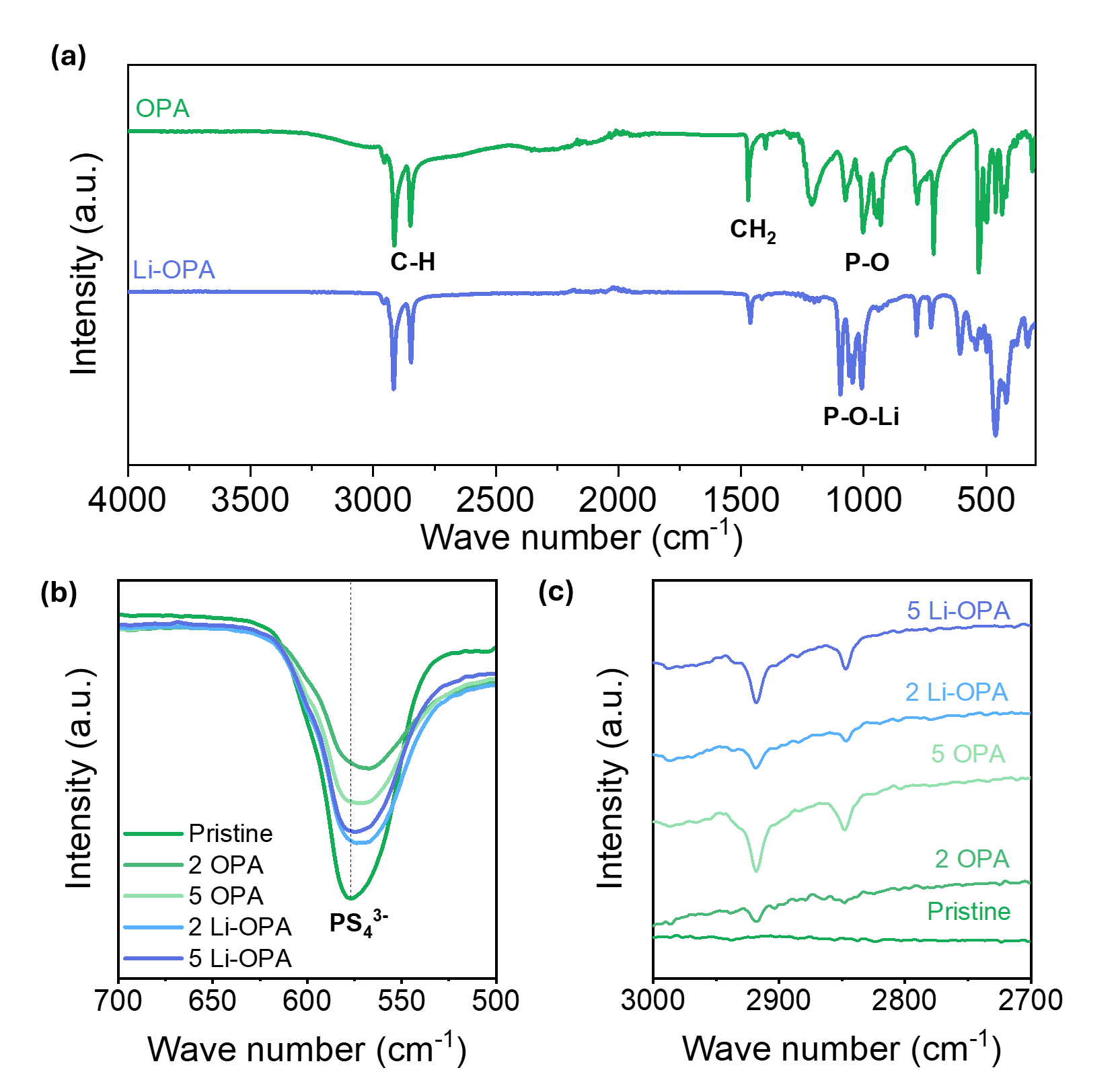


**Figure S1.** FTIR analysis of pristine and organic-coated LPSClBr. FTIR characterization (a) wide spectra, FTIR characterization zoom in on (b) P-S bond region and (c) C-H region of Pristine LPSClBr, OPA-coated LPSClBr, and Lithiated OPA-coated LPSClBr with varying weight percentages.


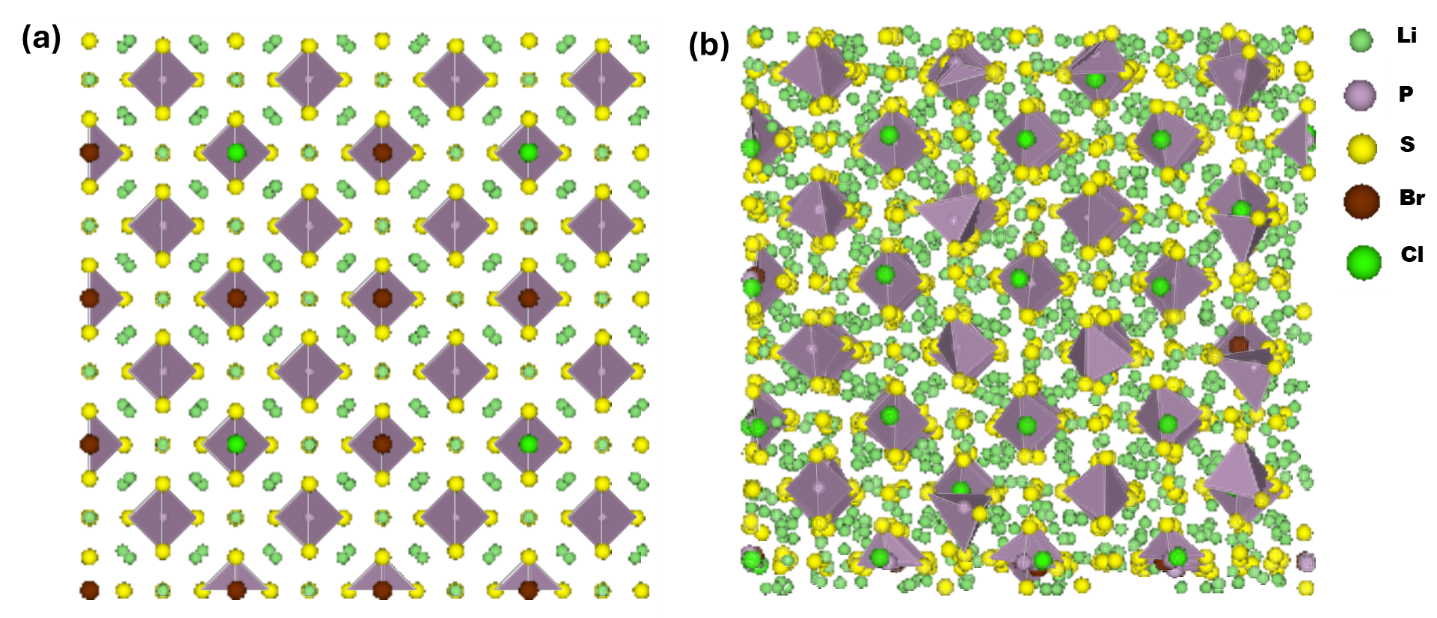


**Figure S2.** LPSClBr surface after a Monte Carlo simulation. (a) Initial configuration of 100 LPSClBr surfaces, with Br atoms initially located at the surface. (b) Final configuration after MC swaps between Cl and Br atoms, showing that the energetically favorable arrangement places Cl atoms at the surface while Br atoms migrate into the bulk.

**
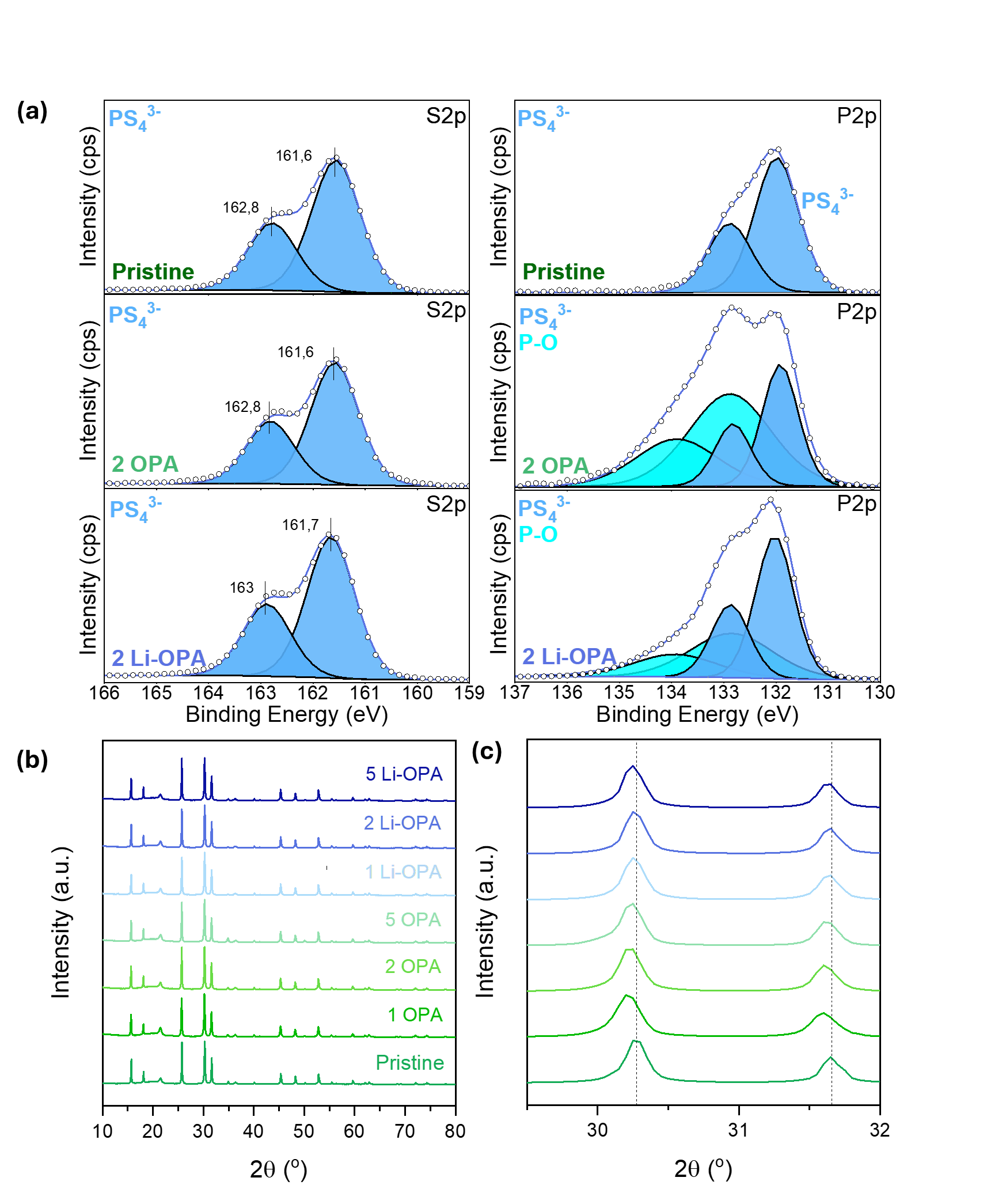
**

**Figure S3.** Structural and surface chemical analysis of pristine and organic-coated LPSClBr via XPS and XRD. (a) XPS spectra of pristine LPSClBr, 2 OPA and 2 Li-OPA solid electrolytes (d) XRD characterization of Pristine LPSClBr, OPA-coated LPSClBr, and Lithiated OPA-coated LPSClBr with varying weight percentages, with (e) corresponding magnification of (311) and (222) peaks.

**
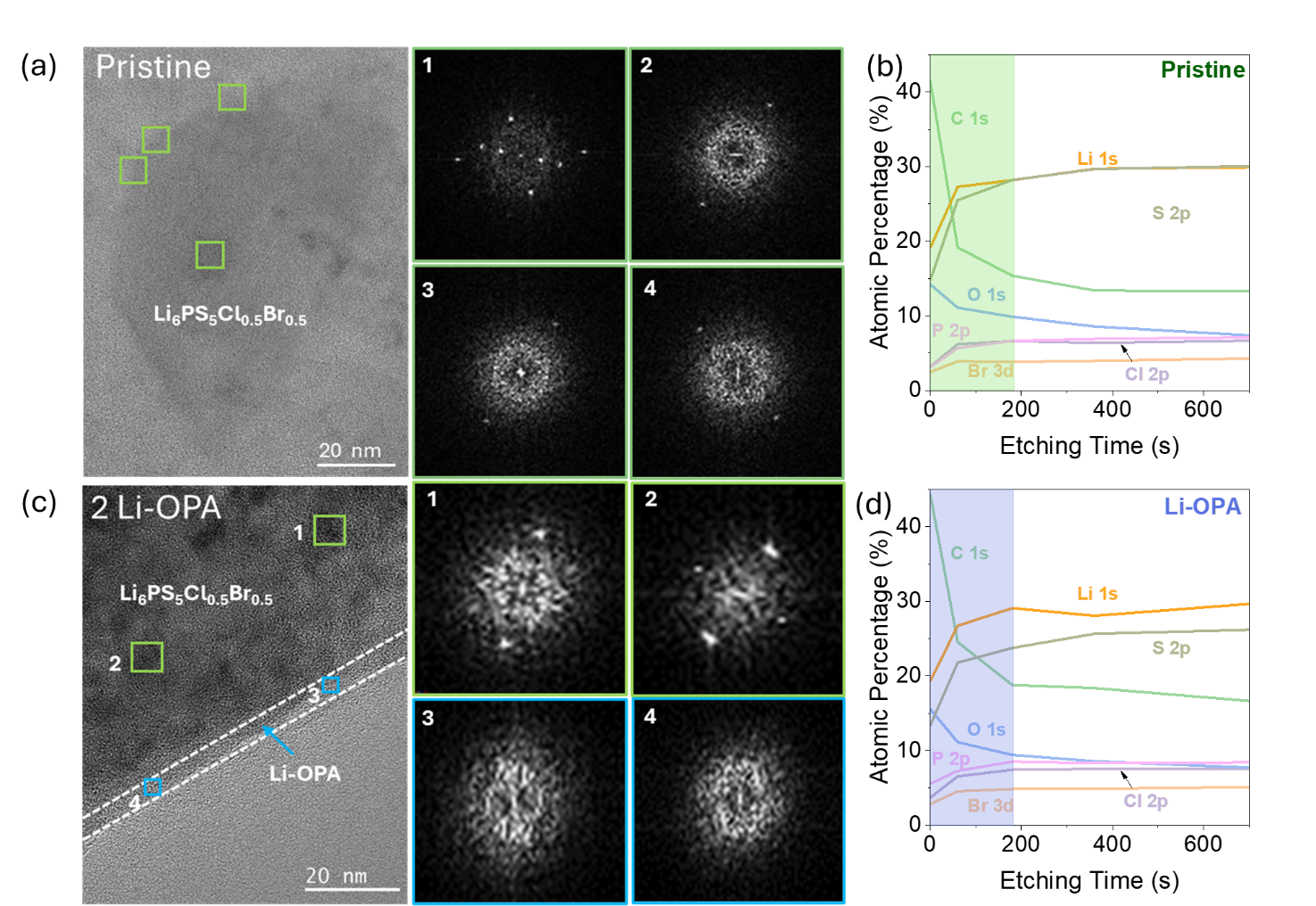
**

**Figure S4.** Coating coverage verified by TEM and XPS depth profile. TEM image of pristine LPSClBr (a) and Li-OPA-coated LPSClBr particles (c), the crystalline domains (Fig.S4c (1,2)) correspond to LPSClBr and the amorphous domains on the surface of LPSClBr correspond to the Li-OPA coating (Fig. S4a (3,4)). XPS elemental depth distribution profiles for pristine (b) and Li-OPA coated LPSClBr (d) samples.


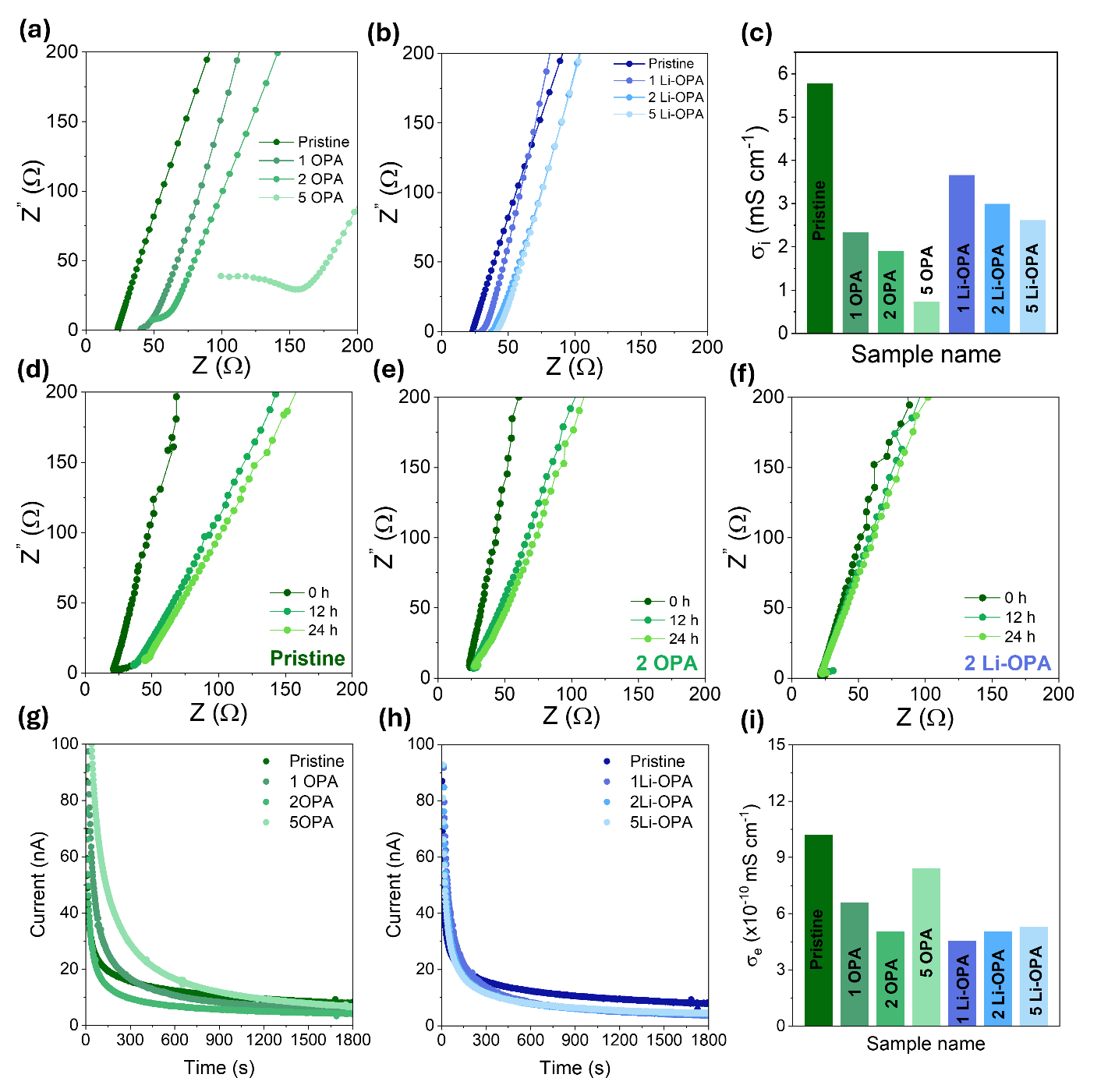


**Figure S5.** Ionic and electronic transport properties of pristine and organic-coated LPSClBr and dry-room stability assessment. Nyquist plot of electrochemical impedance spectroscopy (EIS) measurement (a,b), and the calculated ionic conductivity results of LPSClBr, OPA coated LPSClBr and Li-OPA coated LPSClBr (c). Nyquist plot of EIS measurement (d-f) after exposing the LPSClBr, OPA coated LPSClBr and Li-OPA coated LPSClBr to dry room with dew point -50 °C. for 12 and 24 h The difference in the conductivity of the pristine samples in a and d originates from the batch-to-batch variation of the LPSClBr. (g,h) Direct current polarization and (i) the calculated electronic conductivity results of LPSClBr, OPA coated LPSClBr and Li-OPA coated LPSClBr.


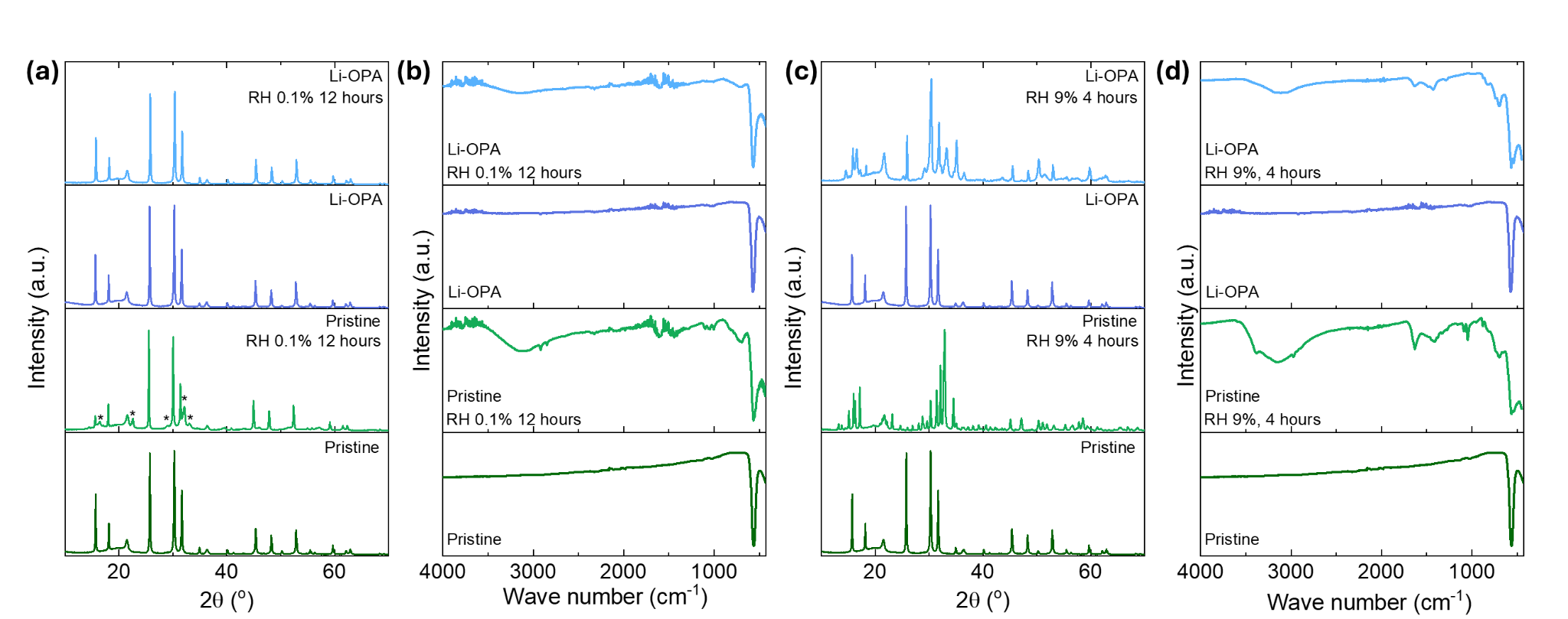


**Figure S6.** Characterization of pristine and 2 wt.% Li-OPA–coated LPSClBr after dry room exposure (-50 °C dew point (0.1% RH, 25 °C), 12 h and -10.1 °C dew point (9% RH, 25 °C), 4 h). (a and c) XRD of pristine LPSClBr and Li-OPA-LPSClBr. XRD patterns reveal that the pristine electrolyte undergoes structural decomposition, while as the coated sample largely preserves its original crystalline features with only the formation of minor secondary phases. (b and d) FTIR of pristine LPSClBr and Li-OPA-LPSClBr. FTIR spectra highlight that both the samples have significant reduction of the PS_4_^3-^ vibrational mode and the pristine sample shows new peaks assigned to OH bonds, Li_3_PO_4_ and Li_2_CO_3_ bonds.


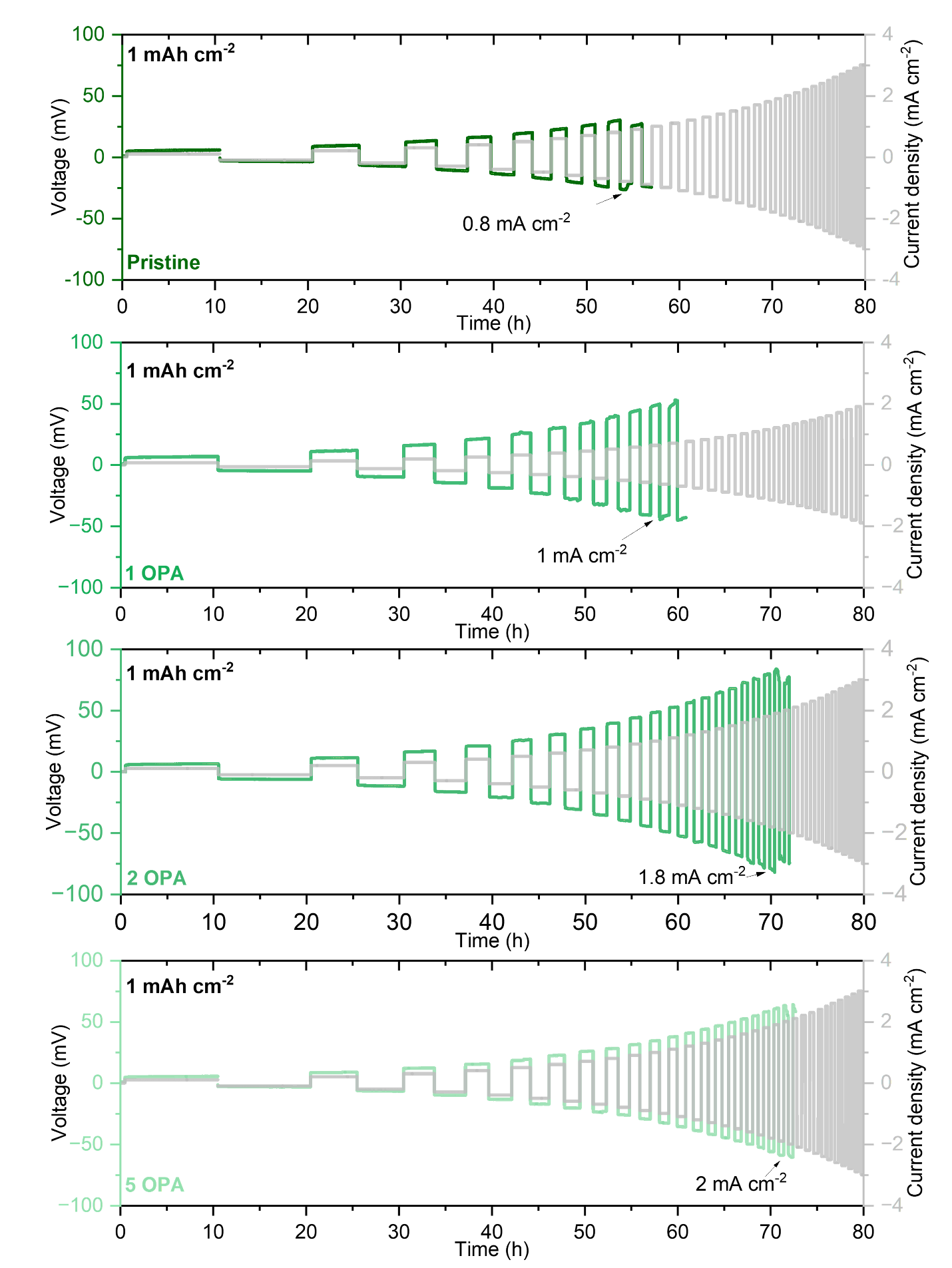


**Figure S7.** Critical current density of Li|Pristine, OPA-coated LPSClBr|Li cells.


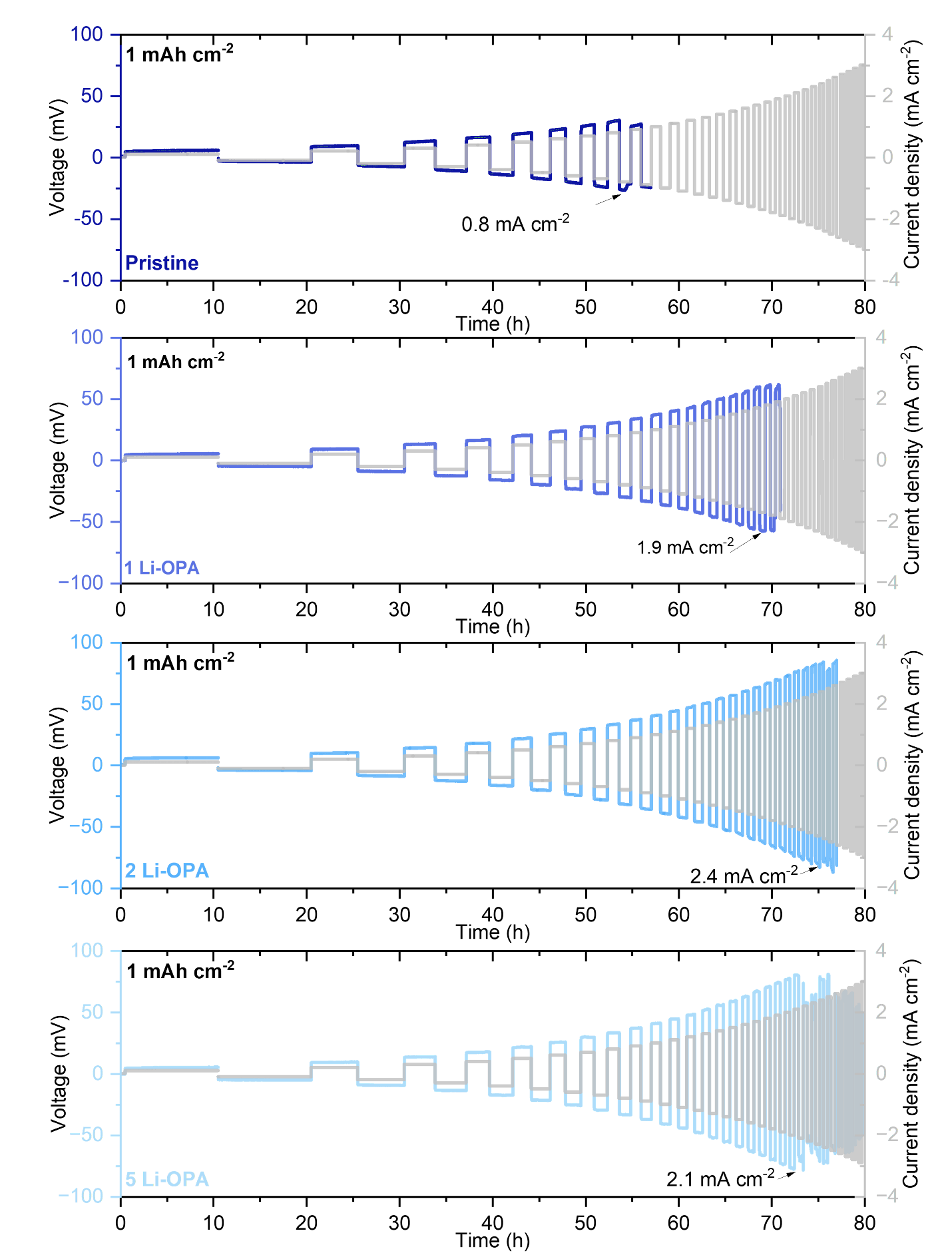


**Figure S8.** Critical current density of Li|Pristine, Li-OPA-coated LPSClBr|Li cells.


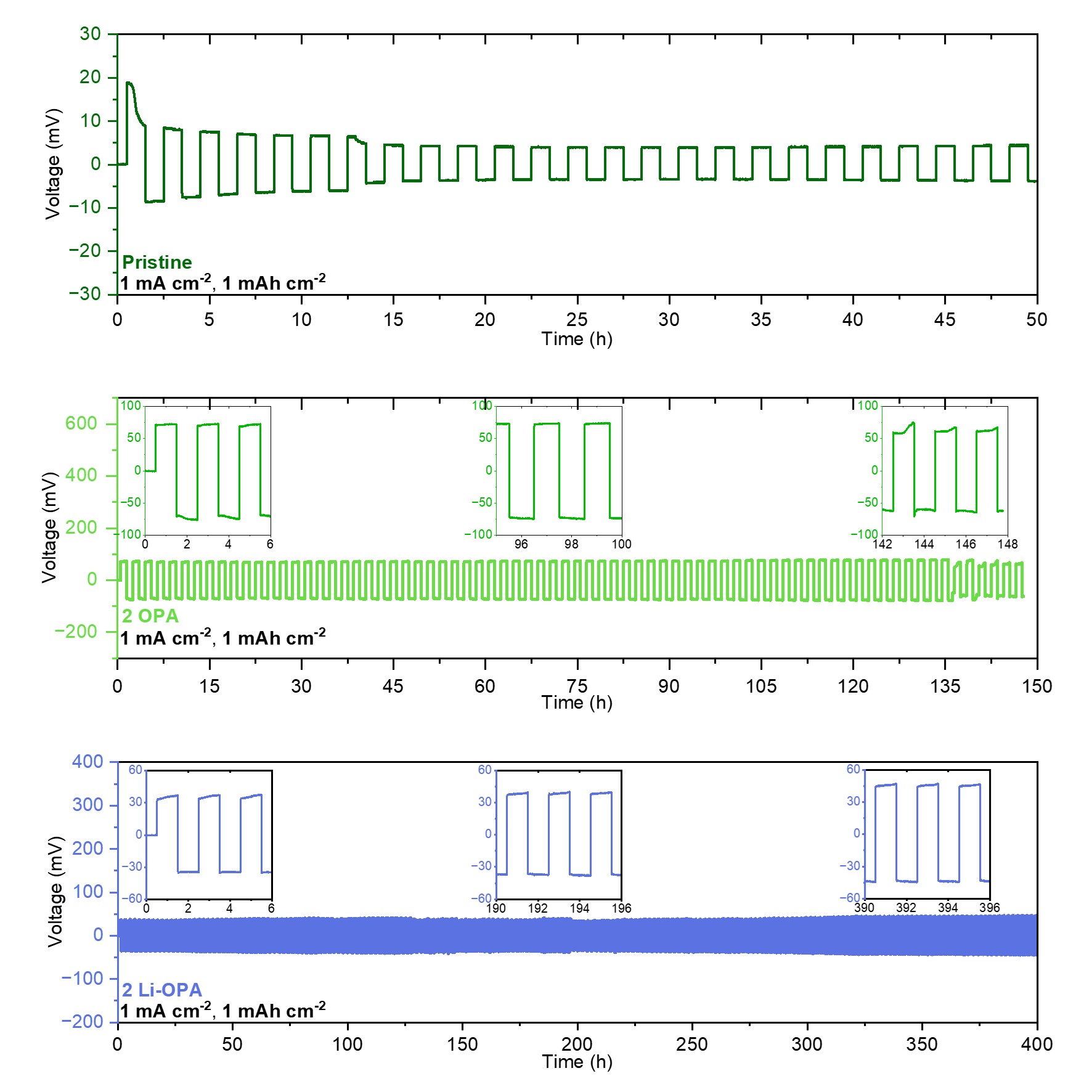


**Figure S9.** Li plating–stripping behavior of symmetric cells with pristine and organic-coated LPSClBr Electrolytes at 1.0 mA cm⁻², 1.0 mAh cm⁻².


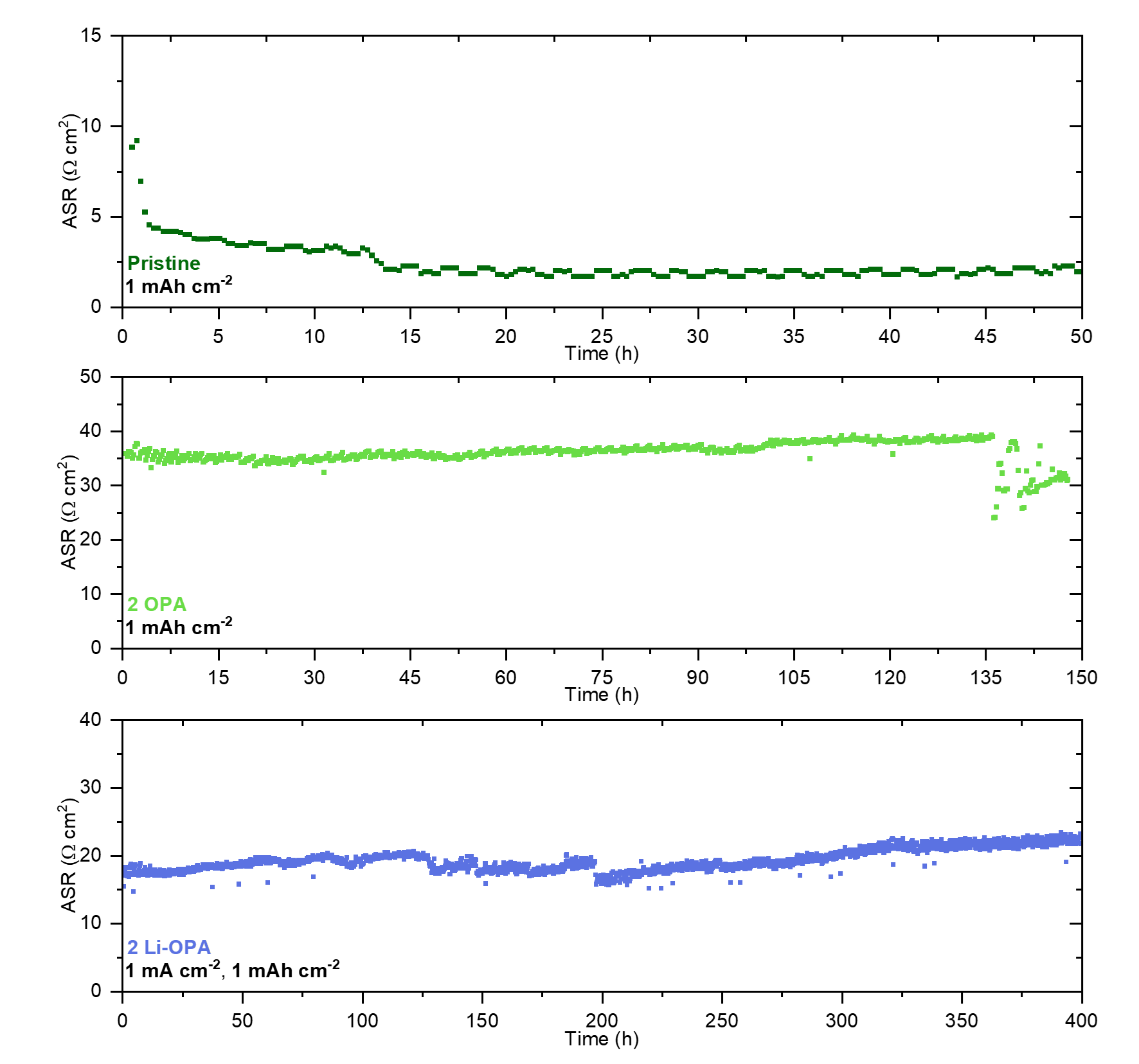


**Figure S10.** The area-specific resistance (ASR) of symmetric cells with pristine and organic-coated LPSClBr electrolytes at 1.0 mA cm⁻², 1.0 mAh cm⁻².


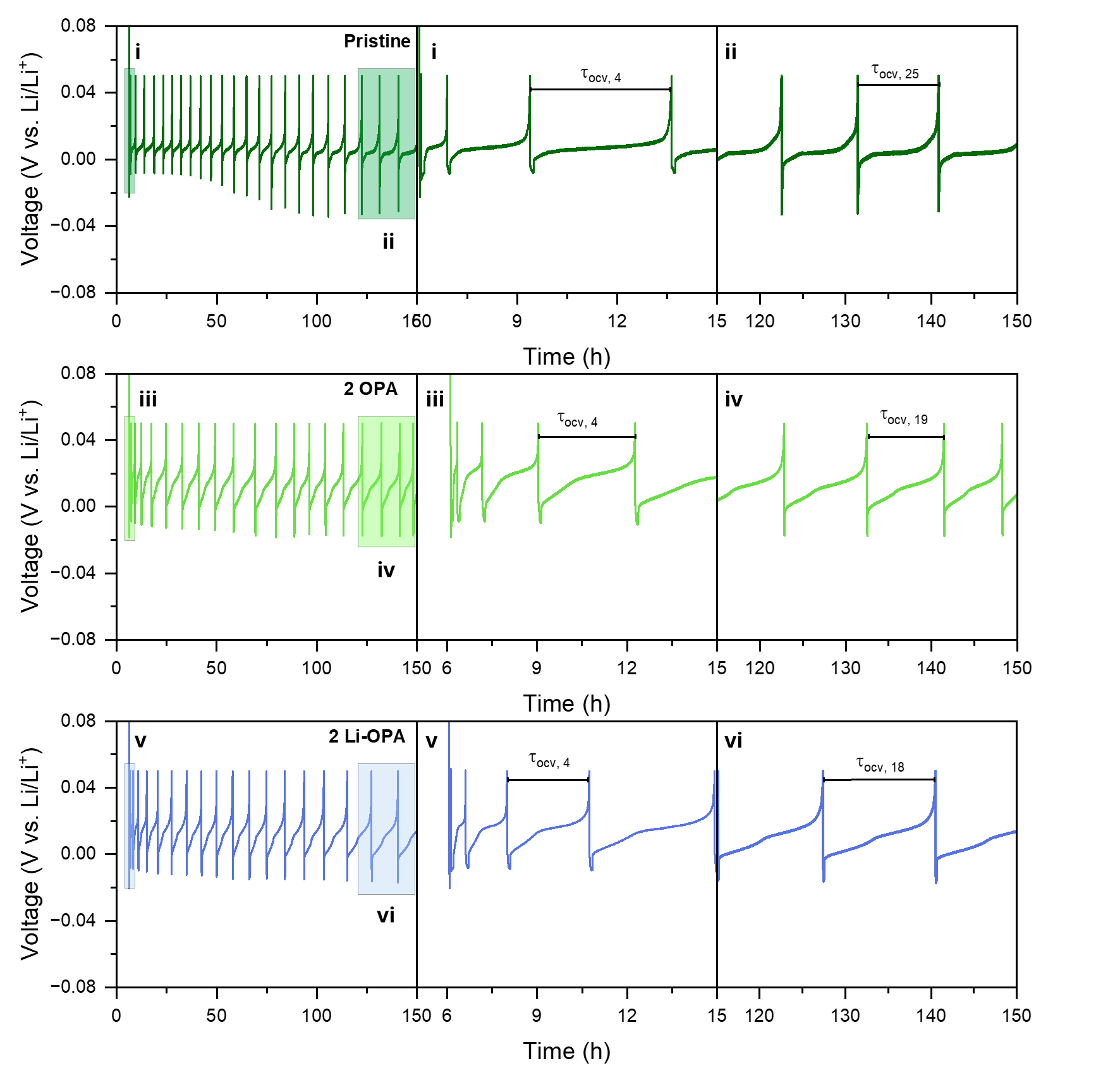


**Figure S11.** CTTA analysis of Ni∣LPSClBr-Coated LPSClBr∣Li cells: potential evolution at initial and extended stages. The potential profiles are presented for selected time intervals, highlighting an early stage and a later stage of the experiment.


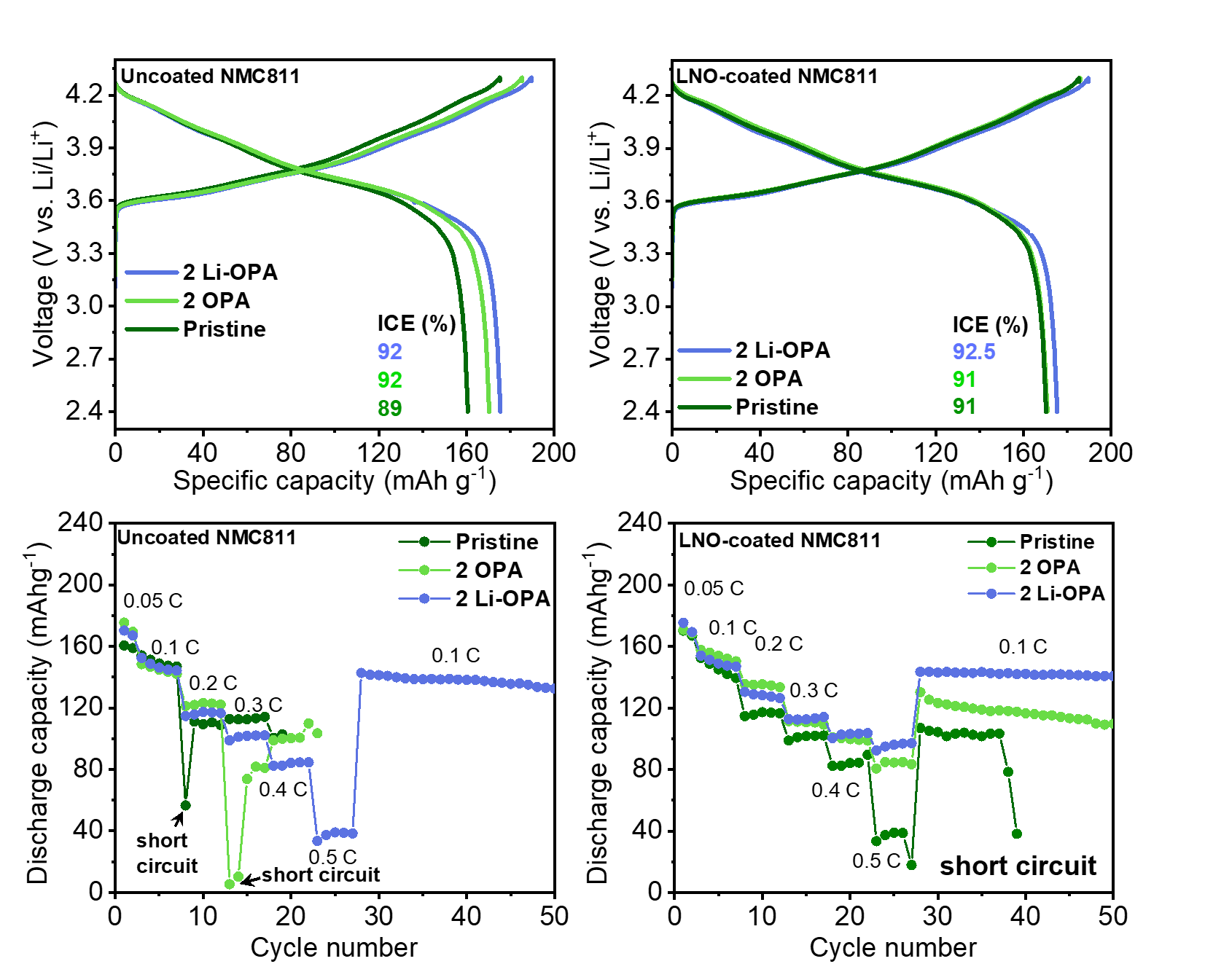


**Figure S12.** Electrochemical Performance of Li|LPSClBr|uncoated and LNO-coated NCM811 ASSBs with pristine and modified electrolytes. First-cycle charge–discharge curves for ASSBs with uncoated (a) and LNO-coated (b) NCM811 cathodes at 0.05 C. Rate performance of cells with uncoated (c) and LNO-coated (d) NCM811 at 9.1 mg cm^-2^.

**
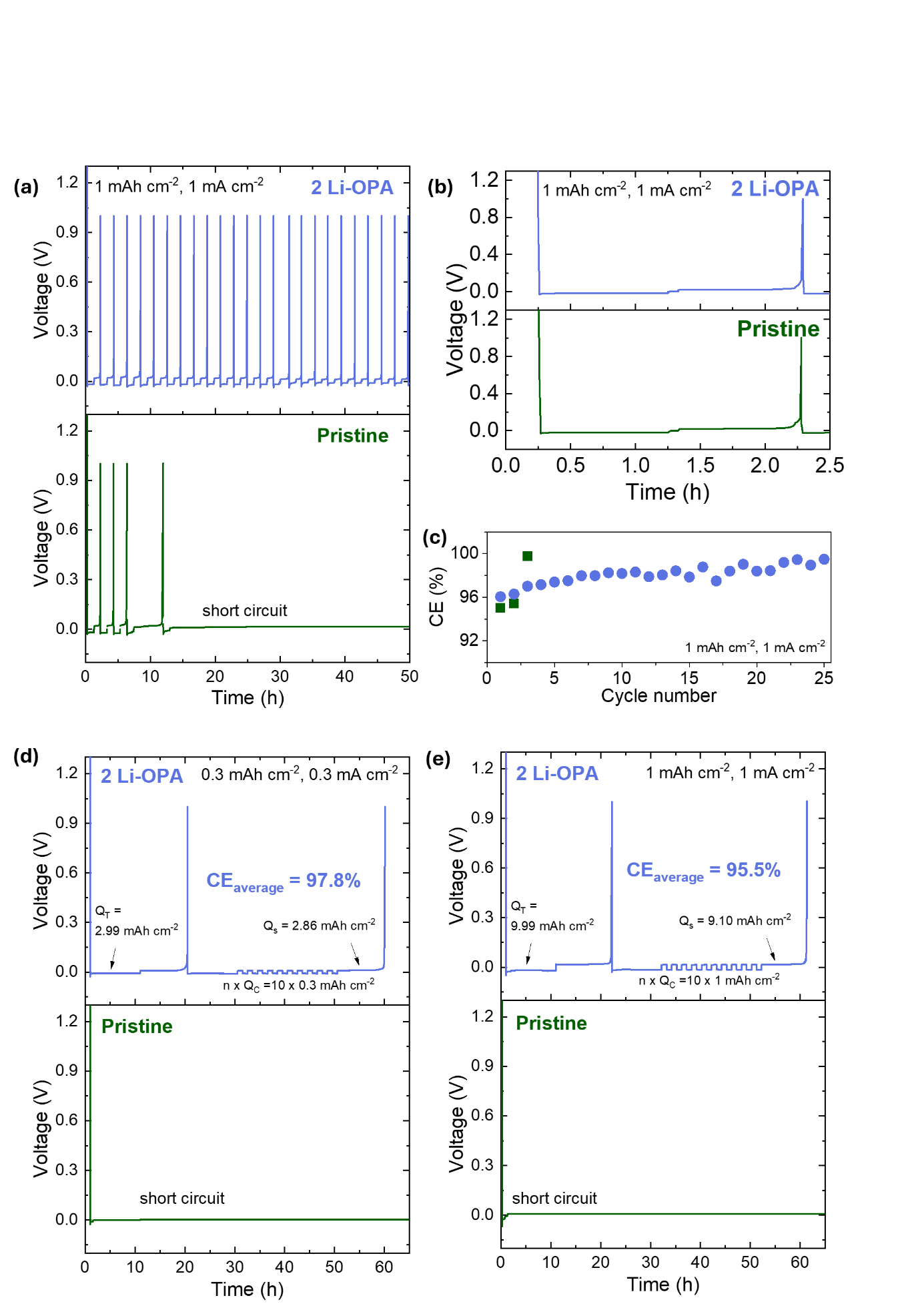
**

**Figure S13.** Stability test of Li–Cu asymmetric cells. (a) Voltage profiles showing short-circuiting of pristine LPSClBr after 3 cycles, while Li-OPA-coated LPSClBr remains stable. (b) First-cycle plating/stripping behavior. (c) Coulombic efficiency evolution over cycling, with Li-OPA-coated samples showing higher and increasing CE. (d,e) Aurbach test results at 0.3 mA cm^-2^ (0.3 mAh cm^-2^) and 1 mA cm^-2^ (1 mAh cm^-2^), respectively.


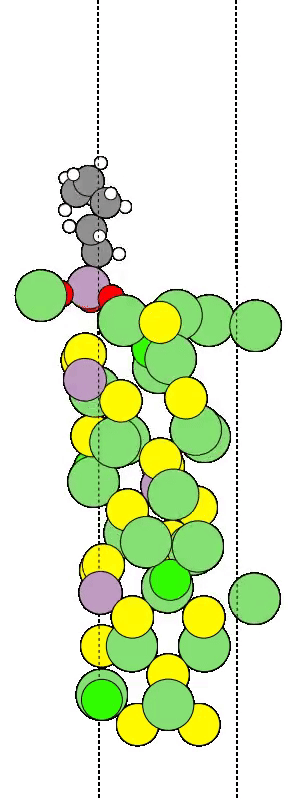

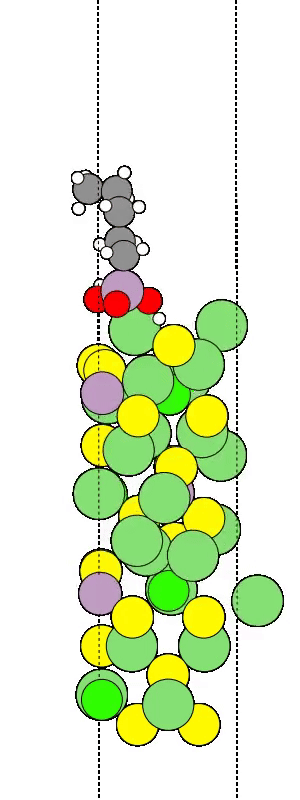


**(b)**

**(a)**

**Supporting Video. 1.** **DFT simulated video of (a) H-OPA and (b) Li-OPA attached to the LPSCl structure**
